# Balancing selection and high genetic diversity of *Plasmodium vivax* circumsporozoite central region in parasites from Brazilian Amazon and Rio de Janeiro Atlantic Forest

**DOI:** 10.1371/journal.pone.0241426

**Published:** 2020-11-09

**Authors:** Natália Ketrin Almeida-de-Oliveira, Rebecca de Abreu-Fernandes, Lidiane Lima-Cury, Aline Rosa de Lavigne, Anielle de Pina-Costa, Daiana de Souza Perce-da-Silva, Marcos Catanho, Atila Duque Rossi, Patrícia Brasil, Cláudio Tadeu Daniel-Ribeiro, Maria de Fátima Ferreira-da-Cruz

**Affiliations:** 1 Laboratório de Pesquisa em Malária, Instituto Oswaldo Cruz (IOC), Fundação Oswaldo Cruz (Fiocruz), Rio de Janeiro, Brazil; 2 Centro de Pesquisa, Diagnóstico e Treinamento em Malária (CPD-Mal), Reference Laboratory for Malaria in the Extra-Amazonian Region for the Brazilian Ministry of Health, SVS & Fiocruz, Rio de Janeiro, Brazil; 3 Laboratório de Pesquisa Clínica em Doenças Febris Agudas, Instituto Nacional de Infectologia Evandro Chagas, Fiocruz, Rio de Janeiro, Brazil; 4 Centro Universitário Serra dos Órgãos (UNIFESO), Teresópolis, Rio de Janeiro, Brazil; 5 Laboratório de Genética Molecular de Microrganismos, IOC, Fiocruz, Rio de Janeiro, Brazil; 6 Laboratório de Virologia Molecular, Departamento de Genética, Instituto de Biologia, Universidade Federal do Rio de Janeiro, RJ, Brazil; University of Iceland, ICELAND

## Abstract

Circumsporozoite protein (CSP) is the primary pre-erythrocytic vaccine target in *Plasmodium* species. Knowledge about their genetic diversity can help predict vaccine efficacy and the spread of novel parasite variants. Thus, we investigated *pvcsp* gene polymorphisms in 219 isolates (136 from Brazilian Amazon [BA], 71 from Rio de Janeiro Atlantic Forest [AF], and 12 from non-Brazilian countries [NB]). Forty-eight polymorphic sites were detected, 46 in the central repeat region (CR), and two in the C-terminal region. Also, the CR presents InDels and a variable number of repeats. All samples correspond to the VK210 variant, and 24 VK210 subtypes based on CR. Nucleotide diversity (π = 0.0135) generated a significant number of haplotypes (168) with low genetic differentiation between the Brazilian regions (F_st_ = 0.208). The haplotype network revealed similar distances among the BA and AF regions. The linkage disequilibrium indicates that recombination does not seem to be acting in diversity, reinforcing natural selection’s role in accelerating adaptive evolution. The high diversity (low F_st_) and polymorphism frequencies could be indicators of balancing selection. Although malaria in BA and AF have distinct vector species and different host immune pressures, consistent genetic signature was found in two regions. The immunodominant B-cell epitope mapped in the CR varies from seven to 19 repeats. The CR T-cell epitope is conserved only in 39 samples. Concerning to C-terminal region, the Th2R epitope presented nonsynonymous SNP only in 6% of Brazilian samples, and the Th3R epitope remained conserved in all studied regions. We conclude that, although the uneven distribution of alleles may jeopardize the deployment of vaccines directed to a specific variable *locus*, a unique vaccine formulation could protect populations in all Brazilian regions.

## Introduction

Malaria is the most prevalent infectious disease globally, with an estimated 228 million cases and 405 thousand deaths in 2018 [1]. Although *Plasmodium falciparum* is responsible for most cases and deaths from malaria, *P*. *vivax* is by far the most widespread, accounting for almost half of non-African malaria cases [2]. More than a third of the world’s population is at risk of infection, posing a severe threat in Asia, Oceania, and South America, where *P*. *vivax* is responsible for about 80% of malaria cases. In Brazil, malaria remains a public health concern, and around 153 thousand cases were recorded in 2019, of which 90% caused by *P*. *vivax* [3].

Brazil presents two autochthonous profiles of malaria transmission: the most important corresponds to the Amazon rainforest, where more than 99% of the malaria cases occur; the second one takes place in non-endemic regions of Atlantic Forest (AF) and represents 0.08% of all malaria cases in Brazil [4, 5]. Although malaria cases have substantially decreased in Southeast Brazil after the malaria eradication campaign in 1960 [4], residual *P*. *vivax* cases persist in AF areas until today, probably as a zoonotic transmission [6]. The singular epidemiological aspect based on the bromeliad-malaria model sustains the autochthonous human cases in the past few decades in Rio de Janeiro state (RJ) [6, 7]. In RJ, the vectors are *Anopheles* mosquitoes, widely found in AF, belonging to the subgenus *Kerteszia*, mainly *An*. *K*. *cruzii*, that utilize bromeliads' retained water as habitats for their immature stages [8]. The *An*. *cruzii*, known for its acrodendrophilic habit, bites monkeys in the canopy and, eventually, humans at ground level. This mosquito behavior makes possible zoonotic malaria transmission in Southeastern Brazil, where monkeys are the parasite reservoir [7–9]. Genetic and morphological similarities between *P*. *vivax*, which infects humans, and *P*. *simium*, which infects monkeys in the Atlantic Forest, is consistent with host switches between them in recent evolutionary times [6, 10–12].

*P*. *vivax* responds slower than *P*. *falciparum* to control strategies due to relapse episodes induced by the dormant liver stage—the hypnozoite–and early emergence of gametocytes soon after the pre-erythrocytic phase, increasing the chances of infecting mosquitoes and humans [4, 13, 14]. Besides that, the increasing number of *P*. *vivax* severe cases [15] and the spread of drug resistance [16] strengthen the priority to vaccine development against vivax malaria [17, 18].

Efforts to develop a pre-erythrocytic vaccine have focused on the circumsporozoite protein (CSP), which is abundantly expressed on the sporozoite surface, and responsible for the motility and invasion of the sporozoite into hepatocytes [19]. CSP-synthetic peptides induced a high and specific humoral response similar to those naturally acquired in endemic areas [20–22]. The structure of CSP is similar among *Plasmodium* species infecting rodents, primates, and humans. The CSP sequence comprises a central repeat region (CR) that is specific to each species and two flanking conserved domains identical among plasmodial species: Region I, located in the N terminal, participates in the binding on the salivary glands of the mosquito and Region II, at C-terminal, is a cell-adhesive motif to the hepatocyte [23]. The repetitive CR biological function of CSP is unknown. The *P*. *vivax* CSP has three types of nonapeptides in tandem that identify the three variants: VK210 (GDRA(A/D)GQPA), VK247 (ANGA(G/D)(N/D)QPG) and vivax-like (APGANQ(E/G)GGAA) [24]. VK210 is the most prevalent and widespread worldwide [25], whereas the VK247 is detected in regions where *P*. *vivax* and *P*. *falciparum* coexist, including Brazil [26–28].

VK247 seems to be more common in South-eastern Asia [29–32]. The vivax-like variant is identical to those described for *P*. *simiovale*, and it had been detected in Papua New Guinea, Brazil, Indonesia, and Madagascar [27, 33, 34]. In Brazil, the vivax-like variant had been disclosed in several Amazonian states [27] and an extra-Amazonian area in the state of Maranhão [34].

Although this molecule may represent a promising vaccine candidate, the *pvcsp* gene polymorphisms may affect the host immune response [34]. Consequently, polymorphism dynamics of circulating genotypes from different geographical areas, even in a low transmission setting, could be an obstacle to the development of effective vaccines against *P*. *vivax* [35–37].

So far, there is no data on *pvcsp* gene diversity in the Atlantic Forest region. Thus, this study aimed to investigate the *pvcsp* polymorphisms in CR (286–825) and C-terminal (826–1,068) nucleotides, in parasites from the Brazilian Amazon, where the vast majority of cases occur, and also in RJ Atlantic forest that presents a particular zoonotic transmission malaria profile.

## Material and methods

### Samples

**Fig 1:**
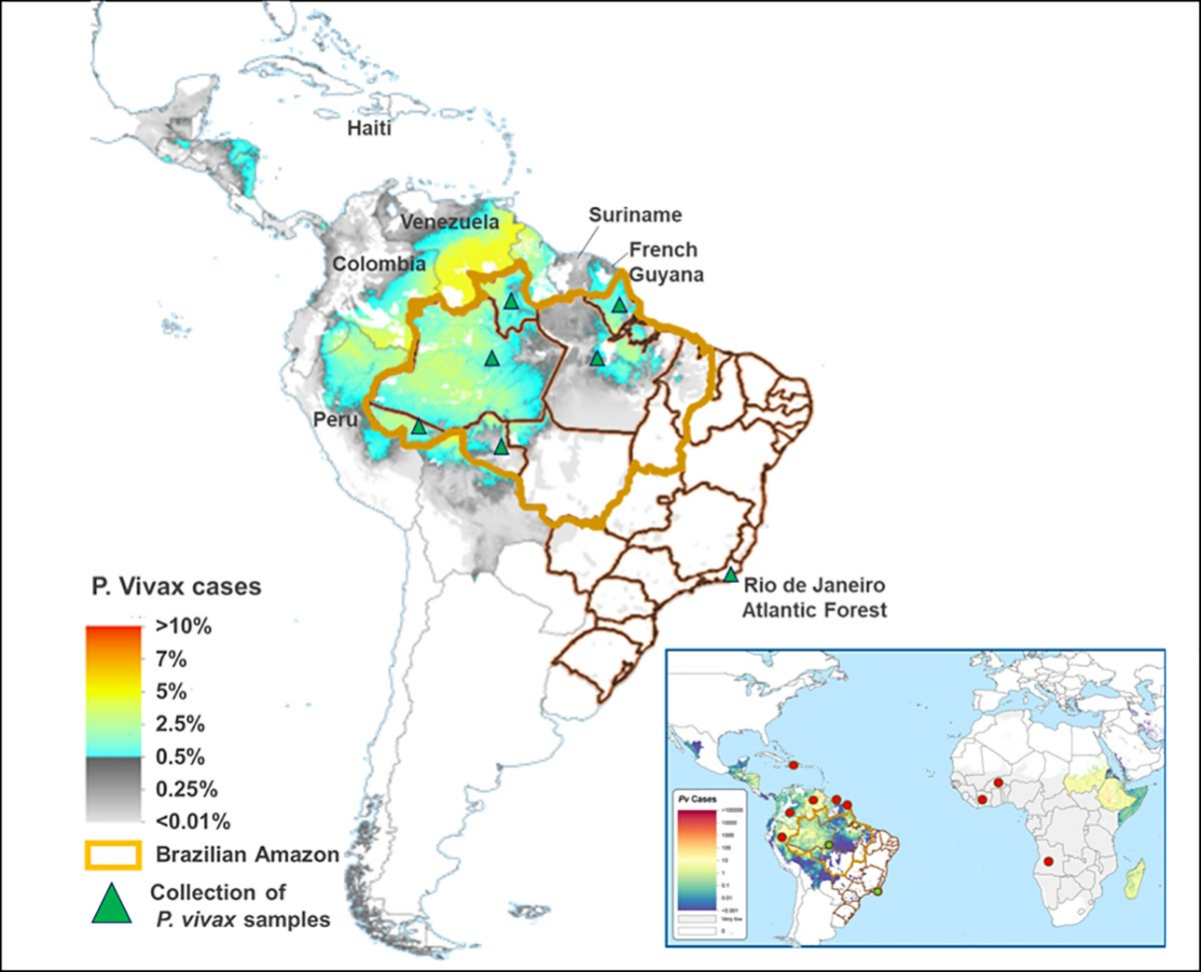
Sampling *P*. *vivax* collection in Brazil and foreign countries. Rio de Janeiro Atlantic Forest and Brazilian Amazon (green). Non-Brazilian samples comprised of *P*.*vivax* isolates from Africa, Central America, and South America (red). This map was generated by the Malaria Atlas Project, University of Oxford (https://malariaatlas.org/). All maps are available under the Creative Commons Attribution 3.0 license (CC BY 3.0; https://creativecommons.org/licenses/by/3.0).

A total of 239 isolates with *P*. *vivax* infection diagnosed by thick blood smear and PCR [38] were tested in this study. Samples were separated according to the site of infection: Brazilian Amazon rainforest (BA), Rio de Janeiro Atlantic Forest (AF), and non-Brazilian (NB) samples (Fig 1). Fifty samples were collected in Tucuruí Unit Health (S 3° 46' W 49° 40'), a municipality in the State of Pará (BA region), while the remaining 189 samples (95 from BA, 82 from AF, and 12 from NB) were collected from patients who have attended a reference center for malaria diagnosis in the Extra-Amazonian region (Centro de Referência para Tratamento e Diagnóstico da Malária—CPD-Mal/Fiocruz) (S 22° 54′ W 43° 12′), between January 2011 and March 2018.

The study was approved by the Ethics Research Committee of Instituto Oswaldo Cruz, Fiocruz, Brazil (69256317.3.0000.5248). All volunteers signed a written informed consent before 5 mL of venous blood collection. The DNA was extracted from 1 mL blood samples using QIAamp™ DNA Blood Midi Kit (QIAGEN, Hilden, Germany), according to the manufacturer’s instructions.

### Primers design and *pvcsp* amplification

The primer set *pvcsp*f 5’ TCCTGTTGGTGGACTTGTTCC 3’ (forward), and *pvcsp*r 5’ GCCAGCACACTTATCCATTGT 3’ (reverse), amplifying a fragment of 1,034 base pairs, was designed using the CPS of Salvador 1 strain (Sal-1; PVX_119355; PlasmoDB: http://www.plasmoDB.org) as a reference, employing the software Primer3web (http://bioinfo.ut.ee/primer3/), and Oligo Analizer™ 3.1 (Integrated DNA Technologies, USA). Amplification specificity was assessed comparing the *pvcsp* primer sequences with CSP nucleotide sequences using the Basic Local Alignment Search Tool (BLAST) algorithm at the National Center for Biotechnology Information website.

The amplification of the *pvcsp* gene fragment was performed in a final volume of 20 μL containing: 2 μL (100–200 ng) of DNA, 1 μM primers (forward and reverse), 5x HOT FIREPol™ Blend Master Mix (Solis Biodyne, Hannover, Germany); 1 mM triphosphate deoxynucleotides (dNTP); 12.5 mM of MgCl_2_ plus enzyme buffer and dyes to increase sample density during the agarose gel electrophoresis. The PCR condition comprised an initial hold (95°C/12 min), followed by 35 cycles (95°C/30 sec, 55°C/45 sec, and 72°C/2 min), and a final hold (72°C/7 min). Negative and positive controls were included in all reactions. PCR products were visualized on ethidium bromide-stained 2% agarose gel electrophoresis under UV transilluminator (DigiDoc-It, UVP, California, USA).

DNA templates were purified using Wizard^™^ SV Gel and PCR Clean-Up System (Promega, Wisconsin, USA), following the manufacturer’s procedure. Subsequently, purified DNA was sequenced using Big Dye^™^ Terminator Cycle Sequencing Ready Reaction version 3.1 (Applied Biosystems, California, USA), with 3.2 μM of forward and reverse PCR primers. DNA sequences were determined using ABI Prism DNA Analyzer™ 3730 (Applied Biosystems, California, USA), at the Fiocruz Genomic Platform PDTIS/Fiocruz RPT01A.

The analysis of the genetic polymorphisms included single nucleotide polymorphism (SNP) and the number of nonapeptide repeats in CR (Insertion and Deletion / InDel).

Nucleotide sequences were aligned using ClustalW multiple sequence aligner in BioEdit software [19], and the electropherogram set to a 10-cutoff score was analyzed using NovoSNP software [39]. The Sal-1 strain was the reference sequence. DNA sequences with singleton mutation or containing overlapped peaks in the same locus were re-sequenced. DNA sequences were deposited in GenBank™ (NIH genetic sequence database; www.ncbi/nlm/nih.gov/GenBank) with accession nos. MN417517-MN417735.

Each nonapeptide repeat in CR corresponds to a “motif”. Each nucleotide sequence of the nonapeptide repeat produced the “allotypes”. The order of repeats at the CR identified the VK210 “subtypes”.

### Genetic diversity, natural selection, and statistical analysis

Genetic diversity of *pvcsp* gene was estimated using the DnaSP 6.11 software [40]. Within-population diversity was measured based on the number of segregating sites (S), nucleotide diversity (π), and haplotype diversity (*Hd*). The distribution of nucleotide diversity was investigated by the sliding window method, in which π is calculated on each window containing a DNA segment of 100 bp with 25 bp overlap.

The variance of allele frequencies between populations was estimated by the Wright’s fixation statistics (F_st_) considering 1,000 pseudo-replicates bootstrapping [41]. To identify sequences that do not fit the neutral theory model at equilibrium between mutation and genetic drift, Tajima’s D test [42] based on the number of segregating sites (θ) was carried out with DnaSP 6.11 software [39].

The relationship between individual genotypes at the population level was assessed through a haplotype network constructed on R with the “haploNet” function of the package “pegas” under an infinite site model [43]. Arguments "size” of “plot” function were attributed to haplotype frequencies, and colors were defined as different regions of occurrence. Each haplotype’s genetic distance was represented by the number of dots on links and was estimated by pairwise differences [44]. The statistical analyses and graphs were performed utilizing GraphPad Prism software version 8.1.2; differences were considered significant with p-value < 0.05.

Linkage disequilibrium (LD) to assess the nonrandom association of alleles located at different loci of the *pvcsp* gene was evaluated for all pairwise combinations by D, D’ and R^2^ using the DnaSP v.5 software and chi-square test. The coefficient of determination R^2^ is the square of the correlation coefficient between two indicator variables. Monomorphic and non-diallelic variants were removed from analyzes. R^2^ for each pair of genetic polymorphisms was plotted on heatmap graphics with R using the “LDheatmap” package [45]. Genetic recombination was calculated using the ZZ statistic (Za—Zns), where larger positive values are expected with increasing recombination [46]. The average of R^2^ overall pairwise comparisons represents Zns [46] and those among adjacent polymorphic sites corresponds to the Za [47].

## Results

### Genetic diversity

PCR products revealed a DNA fragment band ranging from 980 to 1088 bp; unspecific amplifications were not verified. The *pvcsp* gene was successfully amplified in 219 isolates (92%): 71 from AF and 136 from BA, comprising the states of Pará (52), Amazonas (46), Rondônia (17), Acre (11), Amapá (8), and Roraima (2) (S1 Table). Additionally, 12 samples from nine countries spanning three continents were also tested (S1 Table): five from Africa [Burkina Faso (1), Ivory Coast (1), and Angola (3)]; one from Central America (Haiti), and six from South America [Colombia (1), French Guyana (1), Peru (1), Suriname (1), and Venezuela (2)].

**Fig 2:**
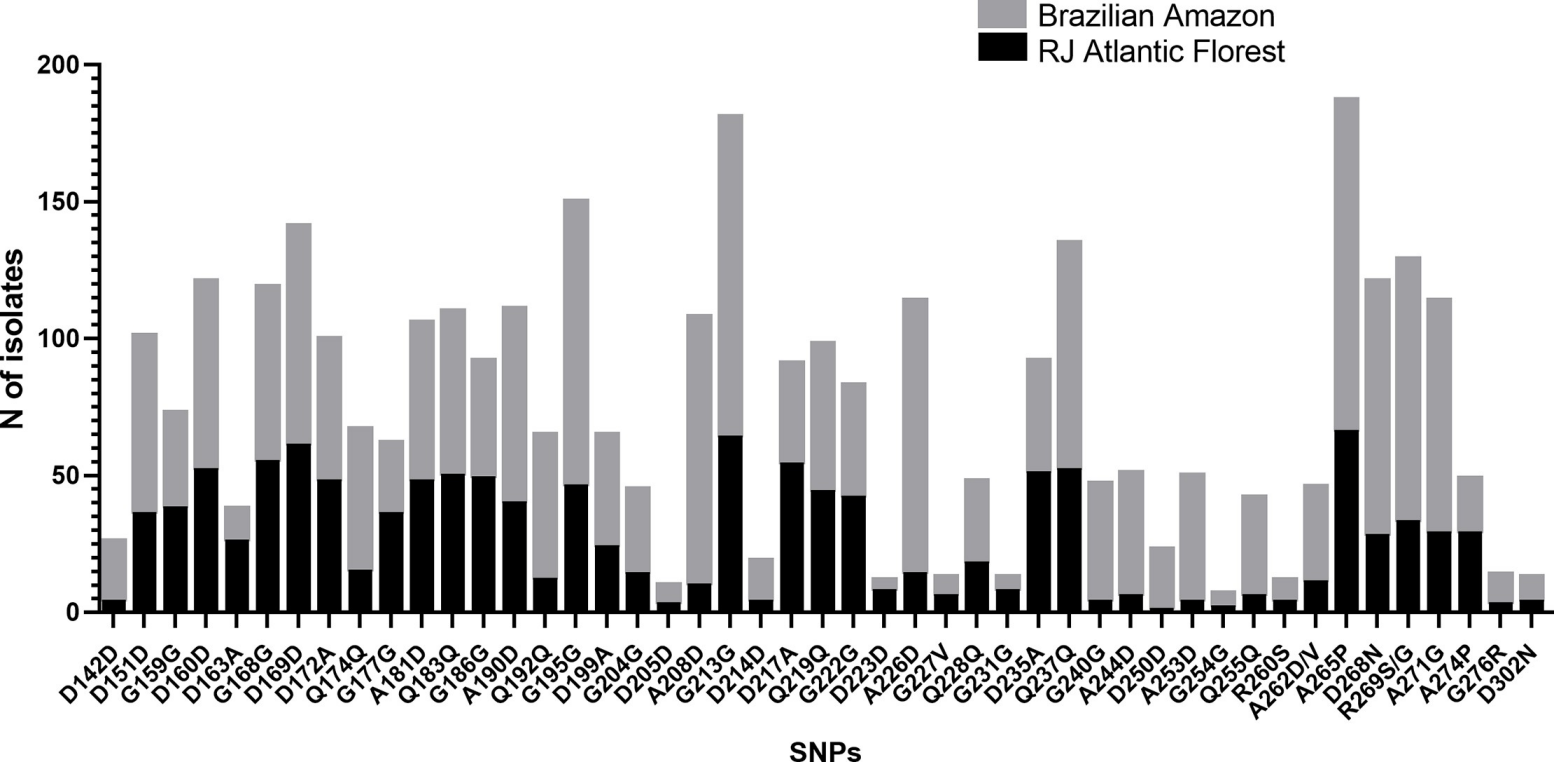
*pvcsp* codons in Brazilian isolates from two geographic regions: AF (71) and BA (136). AF: Rio de Janeiro Atlantic Forest; BA: Brazilian Amazon. The first character represents the amino acid of the Sal-1 reference sequence, followed by the position of this residue (number), and the replacing amino acid observed at the same position (last character). Graph was constructed using GraphPad Prism software version 8.1.2.

Multiple sequence alignment of the amplified *pvcsp*-gene fragment of these 219 isolates disclosed 48 polymorphic sites, 26 synonymous and 22 nonsynonymous distributed in 47 codons (Fig 2 and S2 Table). Most of allelic codons differ by one nucleotide. The R269**S**/**G** codon displayed two nucleotide substitutions (A805**G** and A807**T**) (S1 File) while five codons presented triallelic positions (A504**T**/**C**, T531**C**/**A**, T558**C**/**A**, A612**T**/**C**, and C785**A**/**T**). CR had a higher polymorphic rate (46/96%) than C-terminal. Among the SNPs, seven were detected only in Brazilian isolates: six synonymous (A504**C**, T531**A**, T558**A**, T615**C**, A693**T,** and T750**C**) in CR, and one nonsynonymous (G904**A**) in C-terminal region. All other substitutions were observed at least in one NB sample. (S2 Table). InDels increasing or decreasing the number of nonapeptides from 22 to 18 repeats, respectively, were observed in CR. Insertions were more frequent in AF (38%), and deletions were only detected in BA (4%) (Table 1). The presence of InDels in CR does not seem to be related to those of SNPs in the non-repetitive region (C-terminal), since only one sample from AF presented insertion and nucleotide polymorphism simultaneously.

**Table 1:** *pvcsp* genetic diversity and natural selection in Brazilian and non-Brazilian parasite’s samples.

Diversity							Natural Selection
Region	N	SNP	In/Del	π (SE)	nh	*Hd* (SE)	Tajima's D
**BA**	136	48	22/18	0.0135 (±0.0002)	100	0.99 (±0.002)	2.694 (p<0.05)
**AF**	71	48	22/0	0.0135 (±0.0005)	61	0.99 (±0.002)	1.784
**NB**	12	41	0/18	NA	12	1.00 (±0.030)	0.885

BA: Brazilian Amazon; AF: Atlantic Forest; NB: Non-Brazilian samples; N: number of samples; π: nucleotide diversity; Nh: number of haplotypes; *Hd*: haplotype diversity; SE: standard error; SNP: Single Nucleotide Polymorphism; In/Del: Insertion/Deletion represented by the number of nonapeptide repeats; NA: not applicable.

**Fig 3:**
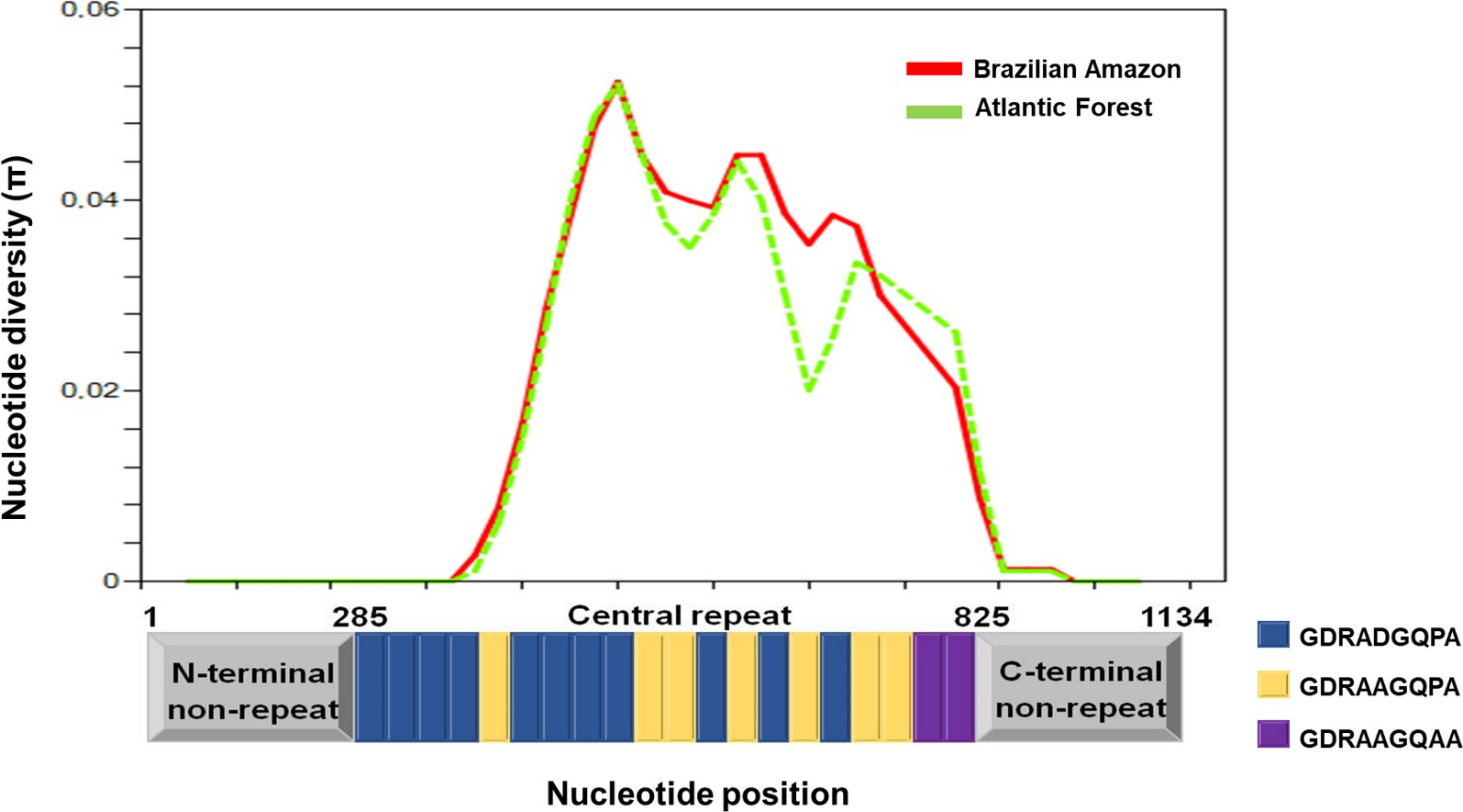
Nucleotide diversity across the *pvcsp* gene in Brazilian isolates. Sliding window representation (window length of 100 bp with a step size of 25 bp) of 136 isolates from Brazilian Amazon and 71 from Atlantic Forest. Sal-1 strain: the *pvcsp* reference sequence; nonapeptide types: 1 (blue), 2 (yellow) and 3 (purple). The π was calculated using the number of SNPs.

The nucleotide diversity in all Brazilian isolates was π = 0.0135 (± 0.0002). Similar diversity was observed when AF and BA regions were analyzed separately, with the highest peak of diversity occurring in CR (nucleotide positions 451 to 550) (π = 0.052) (Fig 3). The great haplotype diversity in Brazilian and non-Brazilian regions was equally distributed: -0.99 for BA and AF-, and 1.00 for NB (Table 1).

The positive value in Tajima’s D test was significant only in BA samples (2.694; p<0.05) (Table 1). These data together with the great nucleotide and haplotype diversities are suggestive of balancing selection.

### Variation in the central repeat region of *pvcsp* gene

**Fig 4:**
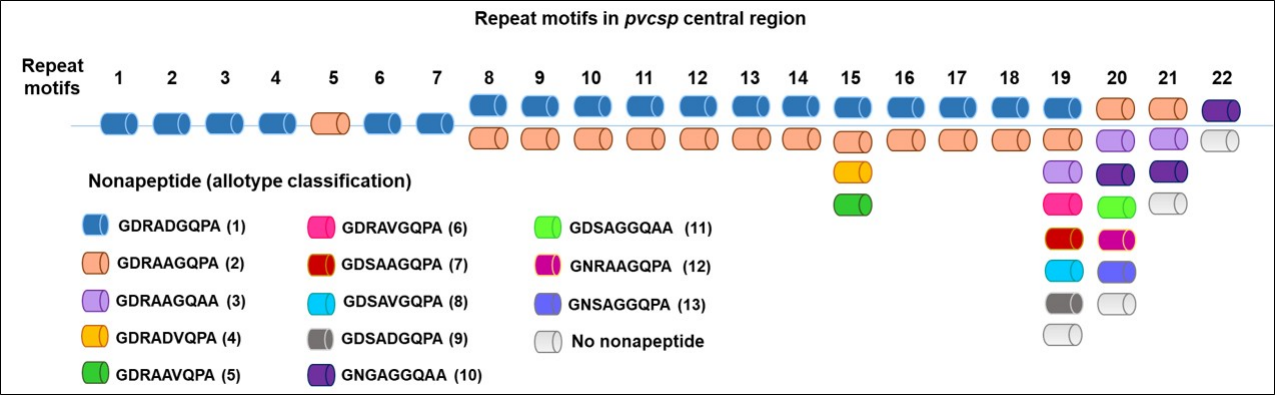
Schematic representation of the nonapeptides in central region of the *pvcsp* gene. Each color represents one of the 13 allotypes, according to the nonapeptide motifs. Motifs 1 to 5 are identical to Sal-1 reference strain; motifs 6 and 7 synonymous SNPs; motifs 21 and 22 insertions and motifs 19 and 20 deletions.

All Brazilian and NB *pvcsp* sequences corresponded to the VK210 type. In CR, the polymorphic sites occurred from the sixth to the twentieth nonapeptide repeats: replacement variations began in the eighth and extended to the last (20^th^) nonapeptide (Table 2 and Fig 4).

**Table 2:** Synonymous and nonsynonymous substitutions in the nonapeptide tandem repeat of central region generating different allotypes.

**Repeat**	**Nonapeptide sequences**	**N of SNPs**	**Nucleotide sequences**
**6**	GDRADGQPA	1	GGA GAT/C AGA GCA GAT GGA CAG CCA GCA
**7**	GDRADGQPA	1	GGA GAC/T AGA GCA GAT GGA CAG CCA GCA
**8**	GDRA(**D/A**)GQPA	3	GGA/T GAC/T AGA GCA G(A/C)T GGA CAG CCA GCA
**9**	GDRA(**D/A**)GQPA	4	GGA/T/C GAC/T AGA GCA G(A/C)T GGA CAG/A CCA GCA
**10**	GDRA(**A/D**)GQPA	3	GGT/C/A GAT AGA GCA G(C/A)T GGA CAA/G CCA GCA
**11**	GDRA(**A/D**)GQPA	3	GGT/C/A GAT AGA GCA G(C/A)T GGA CAG/A CCA GCA
**12**	GDRA(**D/A**)GQPA	2	GGC/A GAT AGA GCA G(A/C)T GGA CAG CCA GCA
**13**	GDRA(**A/D**)GQPA	3	GGA/T/C GAT/C AGA GCA G(C/A)T GGA CAG CCA GCA
**14**	GDRA(**D/A**)GQPA	4	GGC/A GAT AGA GCA G(A/C)T GGA CAG/A CCA GCA
**15**	GDRA(**A/D**)(**G/V**)QPA	5	GGA/T GAT/C AGA GCA G(C/A)T G(G/T)A CAA/G CAA GCA
**16**	GDRA(**D/A**)GQPA	3	GGA/T GAT AGA GCA G(A/C)T GGA CAA/G CCA GCA
**17**	GDRA(**A/D**)GQPA	2	GGA/T GAT AGA GCA G(C/A)T GGA CAG CCA GCA
**18**	GDRA(**A/D**)GQPA	4	GGA GAT/C AGA GCA G(C/A)T GGA/T CAG/A CCA GCA
**19**	GD(**R/A**)A(**A/D/V**)GQ(**A/P**)A	3	GGA GAT AG(A/T) GCA G(C/A/T)T GGA CAG (G/C)CA GCA
**20**	G(**D/N**)(**R/G/S**)A(**A/G**)GQ(**A/P**)A	5	GGA (G/A)AT (A/G)G(A/T) GCA G(C/G)T GGA CAG (G/C)CA GCA

Amino acids and nucleotides underlined represent synonymous substitution; amino acids and nucleotides in bold are nonsynonymous substitution.

**Fig 5:**
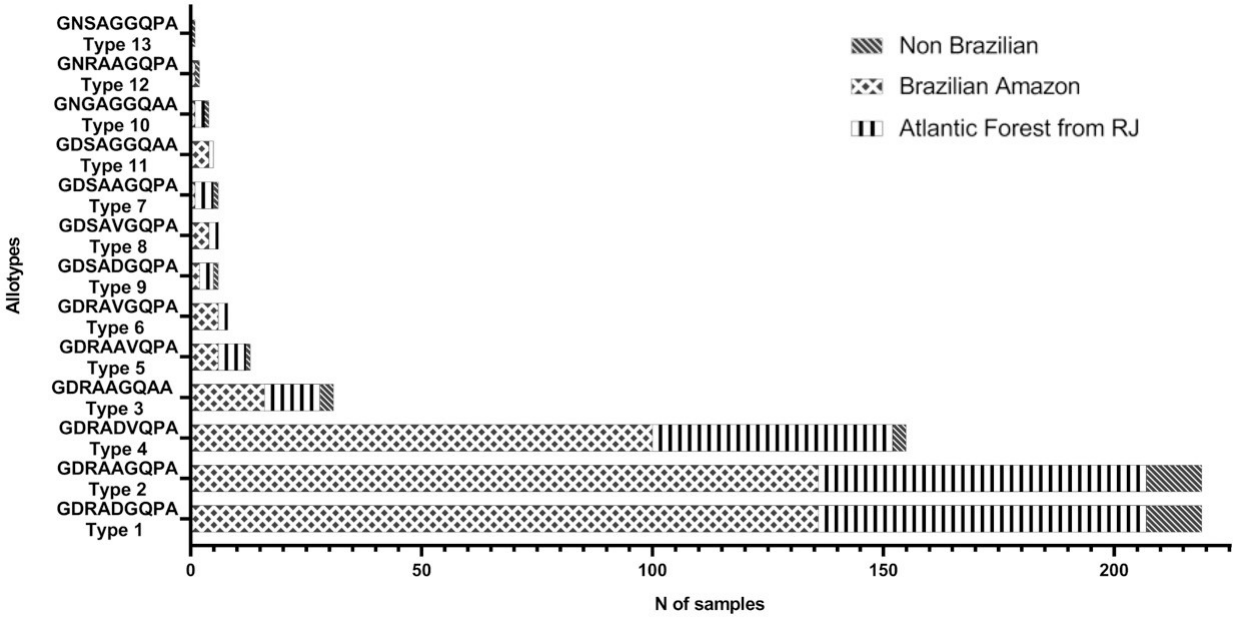
Allotypes distribution of *P*. *vivax* in Brazilian’s and non-Brazilian’s samples.

These SNPs spawned 13 nonapeptides allotypes in the CR. Among them, two nonapeptides—allotypes 1 and 2 –were detected in all isolates (Fig 5). The allotype 1 was the most abundant–GDRADGQPA—varying from seven to 19 repeats, and the type 2 –GDRAAGQPA—from one to 14 repeats. Among the CR motif, three repeats—15, 19, and 20 –presented the largest number of nsSNPs generating 4, 7, and 5 nonapeptide types, respectively (Fig 4 and S3 Table).

Length polymorphism (InDels) at CR presented nonapeptide tandem repeats ranging from 18 to 22; the Sal-1 reference strain had 20 repeats (Table 3). In Brazil, 43 samples (21%) presented InDels: 37 (86%) with insertions and 6 (14%) with deletions. Insertions were observed in 27 samples from AF (38%) and 10 from BA (7%), most of them of Amazonas state (8/ 80%) (S1 Table). Of these samples, 8 (22%) had 21 repeats, and 29 (78%) had 22 repeats, corresponding to an increase of one (type 3: GDRAAGQAA or type 10: GNGAGGQAA) or two (type 2: GDRAAGQPA and; type 10: GNGAGGQAA) nonapeptide repeats. Insertions were not detected in NB.

**Table 3:** Number of InDel polymorphisms in *pvcsp* gene central region, compared to the Sal-1 reference strain’s *pvcsp* gene.

Polymorphism	Number of Repeats	BA (136)	AF (71)	NB (12)	Total
**Insertion**	22	3	26	0	29
	21	7	1	0	8
**Deletion**	19	1	0	0	1
	18	5	0	2	7
**Sal-1 Type**	20	120	44	10	174

BA: Brazilian Amazon; AF: Atlantic Forest; NB: Non-Brazilian; Sal-1: reference strain

Deletions were observed in Brazil only in 6 samples (3%) from BA: one sample had 19 repeats (nucleotides 799–825), and 5 samples had 18 repeats (nucleotides 772–825). In NB, deletions were detected in two samples (17%), one from Suriname and other Venezuela, both with 18 repeats (Table 3, S1 and S3 Tables).

**Fig 6:**
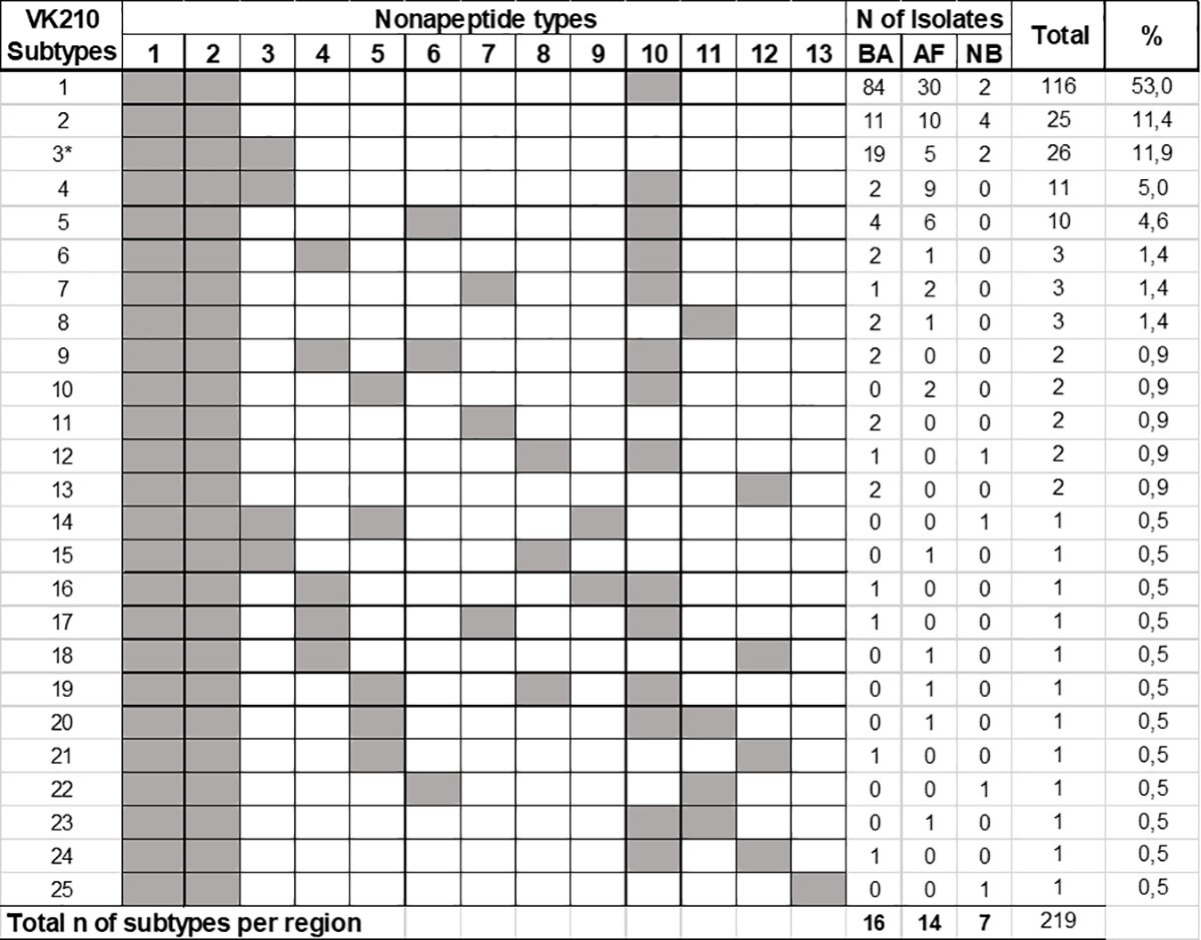
Distribution of VK210 subtypes according to regions. BA: Brazilian Amazon; AF: Atlantic Forest, NB: Non Brazilian; (*) Salvador 1 strain VK210 type; nonapeptide allotypes: 1 (GDRADGQPA); 2 (GDRAAGQPA); 3 (GDRAAGQAA); 4 (GDRADVQPA); 5 (GDRAAVQPA); 6 (GDRAVGQPA); 7 (GDSAAGQPA); 8 (GDSAVGQPA); 9 (GDSADGQPA); 10 (GNGAGGQAA); 11 (GDSAGGQAA); 12 (GNRAAGQPA); 13 (GNSAGGQPA).

Concerning VK210, 24 subtypes were identified (Fig 6). Subtype VK210-1 was the most common, accounting for 53% (116) of the isolates, and the frequencies of the remaining subtypes varied from 5% (11) to 0.5% (one) (Fig 6). Interestingly, 18 VK210 subtypes were found exclusively in Brazilian regions: five (VK210-4-5-6-7-8) in AF and BA, seven only in BA (VK210-9-11-13-16-17-21-24), and six only in AF (VK210-10-15-18-19-20-23). However, the lack of detection of VK210 subtypes in all studied locations may be related to different sampling number among them.

### Distribution of *pvcsp* haplotypes and phylogenetic analysis

**Fig 7:**
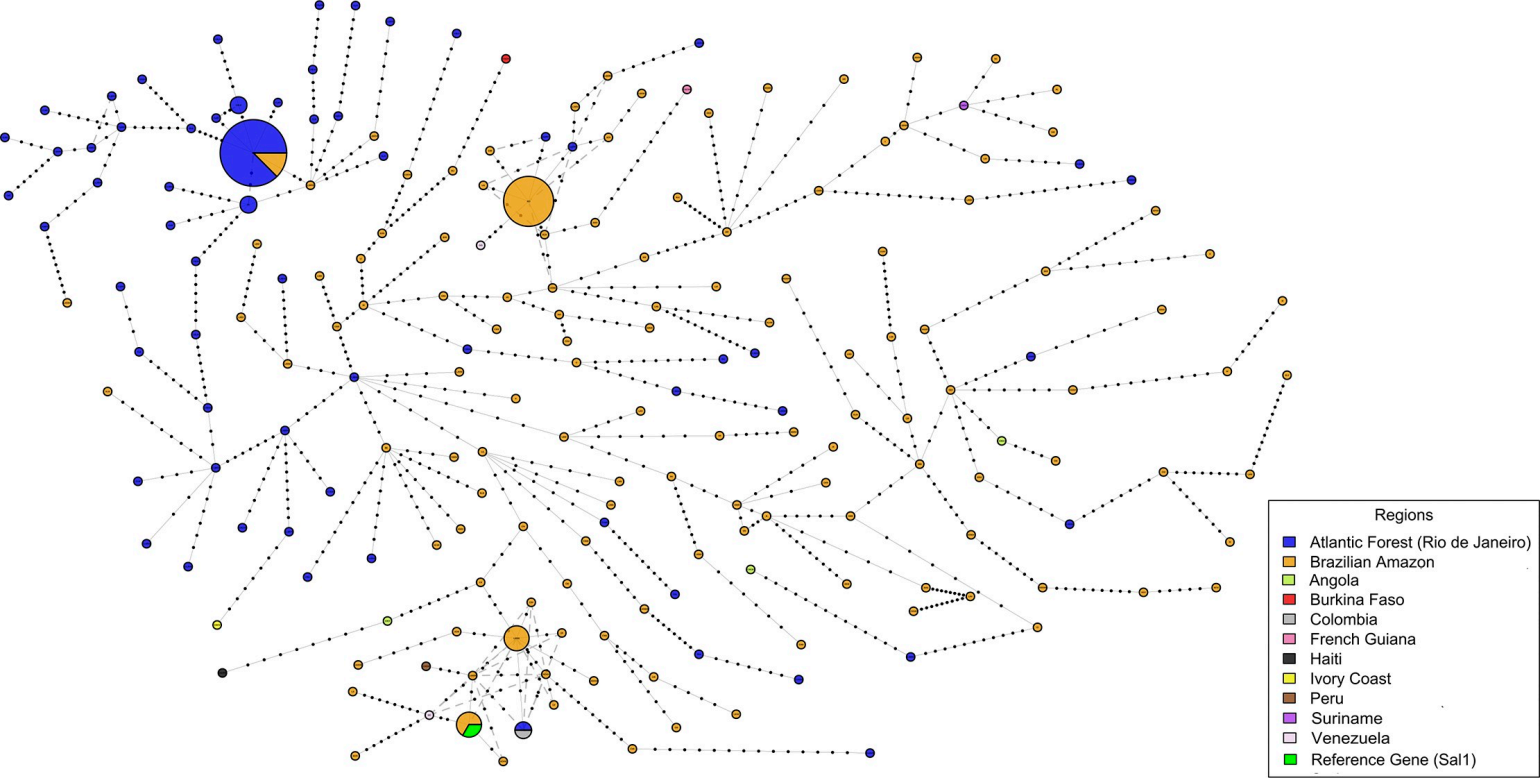
Haplotype network of *pvcsp* gene. Node sizes are proportional to haplotype frequency, and branch lengths are indicative of the number of single nucleotide differences between sequences. Node colors indicate sampling geographic origin.

Considering the arrangement of nonapeptide allotypes of VK210 subtypes and CR nonapeptide repeat length polymorphisms altogether, 168 haplotypes were identified (HCS01 –HCS168). Among them, 100 were detected in BA, 61 in AF, and 12 in NB. Only one isolate (BA/Amapá state) was identical to the reference Sal-1 strain (Fig 7 and S3 Table).

Four Brazilian haplotypes were also found in NB samples: HCS03 (Amazonas, Pará, Rondônia and Angola); HCS11 (AF and Colombia), HCS15 (Amapá and Peru), and HCS19 (Pará and Suriname); only one Brazilian haplotype—HCS01 –was detected in AF and BA. All other 163 haplotypes were segregated according to BA, AF, and NB localities (Fig 7 and S3 Table). Ninety-six out of 100 BA haplotypes were exclusively detected in this area, and among the 61 AF haplotypes, 59 were exclusive of this region. In NB samples eight out of 12 haplotypes were not detected in Brazilian samples: Angola (HCS51 and HCS80), Ivory Coast (HCS22), Burkina Faso (HCS86), Haiti (HCS62), French Guyana (HCS101), and Venezuela (HCS67 and HCS110).

The haplotype network tree showed that BA samples were closely related to Sal-1 reference sequence (Fig 7). Low genetic differentiation, consistent with a weak population subdivision, was observed between AF and BA populations (F_st_ = 0.208/ p<0.001).

### Linkage disequilibrium

**Fig 8:**
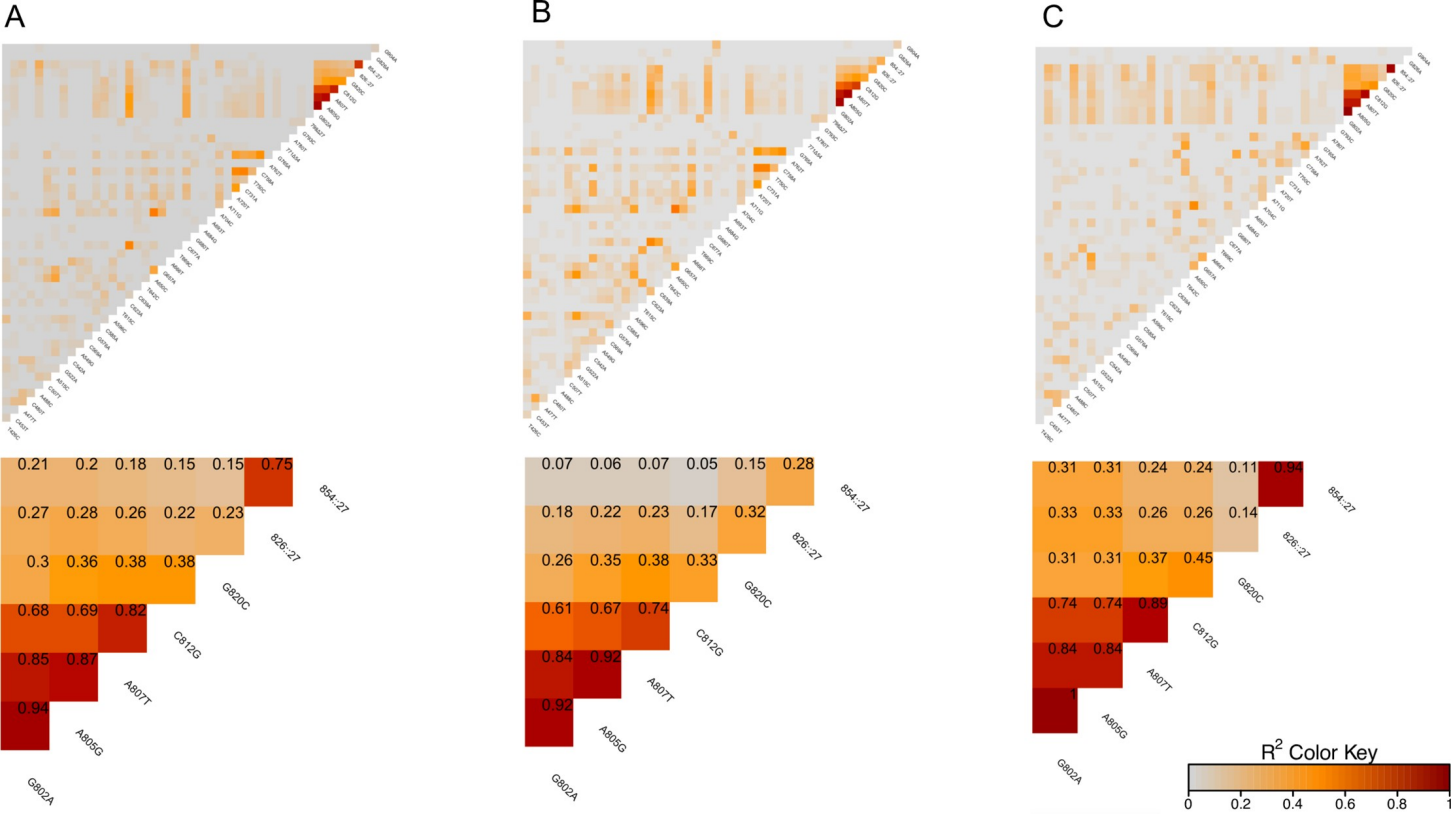
Linkage disequilibrium across the *pvcsp* gene of parasite populations from Brazilian Amazon and Rio de Janeiro Atlantic Forest. A: Parasites from Brazilian Amazon and Rio de Janeiro Atlantic Forest; B: Brazilian Amazon; C: Rio de Janeiro Atlantic Forest; 771Δ54 deletion of 2 repeats; 798Δ27 deletion of 1 repeat; 826::27 insertion of 1 repeat; 854::27 insertion of 2 repeats.

Linkage disequilibrium was assessed for all possible pairwise combinations. The results considering all samples, showed an overall low occurrence of LD. Higher signals (R^2^ ≥ 0.75) were observed between G802**A**, A805**G,** and A807**T**, suggesting a small LD block containing these three polymorphisms. High LD levels were also found between A807**T**/C812**G** and between insertions 826::27/854::27 (Fig 8A). When split by regions, results from BA showed high levels of LD only between G802**A**, A805**G,** and A807**T**, while AF samples displayed a large LD block including G802**A**, A805**G**, A807**T,** and C812**G**. Perfect LD (R^2^ = 1) was found for G802**A** and A805**G**, and almost perfect for 826::27/854::27 insertions in AF samples (Fig 8B and 8C). The ZnS, Za, and ZZ had low values across the *pvcsp* sequence, suggesting that meiotic recombination has not played a crucial role in shuffling nucleotide variation among DNA sequences (Table 4).

**Table 4:** Linkage disequilibrium test across the *pvcsp* sequence by ZnS, Za, and ZZ statistics.

	AF	BA	Total
**Za**	0.125	0.072	0.060
**Zns**	0.047	0.054	0.038
**ZZ**	0.078	0.018	0.022

### Polymorphism in B- and T- cell epitopes

When analyzed four B-cell epitopes [19, 20], no SNPs were found in N- and C- terminal regions coding peptides P8 (GDAKKKKDGKKAEPKNPREN), P24 (CSVTCGVGVRVRRRVNAANK) and P25 (VRRRVNAANKKPEDLTLNDL), but P11 (GDRADGQPA; allotype 1), in CR, presented a nsSNP in five of the nonapeptide repeats (motifs 8, 9, 12, 14 and 16) generating, therefore, allotype 2 (Table 5).

**Table 5:** Polymorphisms in B- and T-cell epitope regions of *pvcsp*.

Peptide	Residues	Position	Epitope	n of SNPs	n of isolates
**Th2R**	PNEKSVKEYL(D/N)KVRATVG	292–309	T-helper	1 NS	13
**P1**	YL(D/N)KVRATV	300–308	T-cytotoxic	1 NS	13
**P11**	GDRA(D/A)GQPA	96–104	B	4 (3S / 1NS)	219
**P15** (motif 19)	GD(R/A)A(A/D/V)GQ(A/P)A	258–266	T-helper	3 NS	201
**P15** (motif 20)	G(D/N)(R/G/S)A(A/G)GQ(A/P)	267–275	T-helper	4 NS	177

Epitopes previously identified [19, 20, 33, 48, 49]; NS: nonsynonymous; S: synonymous.

In relation to the three TCD8^+^ cytotoxic cell epitopes (P1, P3 and P5) [48] located in the C-terminal region, only P1 presented a nsSNP [YL(D/N)KVRATV] in 6% (13) of the Brazilian samples: 7% (9) from BA and 6% (4) from AF (Table 5).

Considering now the three TCD4^+^ helper cell epitopes (P6, P15 and P25) [19, 49], only P15 (GDRAAGQAA/allotype 3), located in CR, was polymorphic. Of the 219 samples, only 39 (18%) presented the P15 epitope sequence conserved but the number of repeats varied from 1 to 3: six samples, three from BA and three from AF, had two repeats, as Sal-1 reference strain; two samples from BA had three repeats and; 31 samples, 16 from BA, 12 from AF and 3 from NB had one repeat. In relation to the polymorphic sequences at CR, the P15 epitope presented in motif 19 three nsSNPs [GD(R/A)A(A/D/V)GQ(A/P)A] in 201 (92%) samples. The motif 20 disclosure four nsSNPs [G(D/N)(R/G/S)A(A/G)GQ(A/P)A] in 177 (81%) samples (Table 5).

In the Th2R epitope [PNEKSVKEYL(D/N)KVRATVG] [33], located in the C-terminal region, a nsSNP was identified in 13 (6%) of Brazilian samples: 4 (6%) from AF and 9 (7%) from BA. No polymorphisms were detected in Th3R epitope.

## Discussion

*pvcsp* is a single copy gene located in chromosome 8 of *P*. *vivax*. It is highly expressed on sporozoites’ surface, playing an essential role in adhesion and invasion of hepatocytes [50]. Due to its surface localization, along with the antibody protective response, this protein has been considered as a malaria pre-erythrocytic phase vaccine candidate [51]. The knowledge regarding the nature and extent of *pvcsp* genetic diversity in Brazil is limited since the majority of *pvcsp* studies had been focused on the immunological response triggered by CSP epitopes [22, 52] or the characterization of *pvcsp* variants associated with the response to treatment, or even in the detection of such variants in *Anopheles* mosquitoes [26, 34]. Now, we report for the first time the genetic diversity, linkage disequilibrium, and natural selection of *pvcsp* gene in two different epidemiological sites, the Brazilian Amazon rainforest (BA) and the Rio de Janeiro Atlantic Forest (AF).

Only *pvcsp’s* gene variant VK210 was detected in both BA and AF; consequently, no association with the presence of VK210 and the geographic origin of the parasites could be made. Although other two variants—VK247 and vivax-like—have already been detected in *P*. *vivax* parasites from the Brazilian Amazon [22, 26, 28, 53] and pre-Amazon [34], the cosmopolitan variant VK210 also predominate in French Guyana [54], Nicaragua [55], Pakistan [30], Sri Lanka [56], Myanmar [57], Cambodia [29], Iran [58], Vanuatu Island [59], and China [25].

It has been claimed that the presence and extension of *pvcsp* variants differ according to transmission intensity [60]. However, this study and those performed in countries with different transmission intensities, where only VK210 type was detected, as Sudan, Azerbaijan, Sri Lanka [61], Honduras [62], and China [63], do not support this proposition. Additionally, the fixation of this variant in Brazil, and perhaps around the world, seems not to be associated with mosquito vectors infection, since malaria transmission in BA occurs mainly by *An*. *Darlingi* and *An*. *albitarsis*, while in AF malaria transmission occurs almost exclusively by *An*. *(Kerteszia) cruzii* vector [7]. Altogether, these findings indicate that VK210 may be the best-adapted variant globally, possibly, due to the more successful evasion of such *P*. *vivax* parasite variant from the immune system.

Sequence analyses revealed high CR diversity, contrasting to none or low diversity in the N- and C- terminals (5’ and 3’ ends), respectively. Indeed, the pre-central (Region I) and the post-central (Region II) repeat sequences that contain both B- and T-cell epitopes [64] remained conserved in this study. The motif known as Region II was also found conserved in previous studies on the Brazilian Amazon [65], Peru [66], Iran, Sri Lanka, India [67], Cambodia, and Colombia [29]. This constrained variation in terminal regions reinforces such protein structures importance for its interaction with *Anopheles* mosquito salivary gland and human hepatocytes during the invasion processes [52]. Conversely, CR diversity could reflect the host’s immune system’s evasion mechanism, as already observed for other surface antigens as *pvdbp* gene [12, 68].

A large number of VK210’s substitutions and length polymorphisms have been found worldwide [26, 69]. However, its nonapeptide repeat structure remained conserved, probably by intragenic shuffling, as previously suggested for *P*. *falciparum* parasites [70]. In this study, all *P*. *vivax* sequences contain one or more copies of two nonapeptide motifs displaying three synonymous substitutions (allotypes 1 and 2). The persistence of these two common allotypes suggests its maintenance by selective pressure. These silent polymorphisms could protect the parasites against stochastic reductions in variability, such as those resulting from bottlenecks. On the other hand, the allotype 3, considered a T-helper cell epitope, revealed an “excess” of nonsynonymous substitutions in the great majority of the samples. The low Sal-1 allotype 3 frequency has already been reported in China [63], Mexico [69], Sudan [61], and also in Brazil [27, 28, 71]. Thus, it is reasonable to speculate that such nonsynonymous polymorphisms are not essential for *P*. *vivax* parasites’ survival, but they might help to evade the host immune response.

The allotype 10 (GNGAGGQAA), also present in Belem 1 strain (GenBank: EU401923), a Brazilian reference strain, was detected in high frequency (71%) mostly in BA isolates. Other investigations in Korea [72], Cambodia [29], Vanuatu [59], and Iran [58] also revealed the presence of this allotype, demonstrating its global distribution. In contrast, among the other nine low-frequency allotypes, only one—allotype 12 (GNRAAGQPA)–was already reported in Sri Lanka [56] and Cambodia [29]. The low prevalence of these novel VK210 subtypes might suggest a relatively recent introduction and/or expansion of these genotypes into Brazilian territory, as previously observed in Iran’s malaria transmission areas [58]. Inversely, allotype 13 (GNSAGGQPA), only detected in French Guyana, suggested that this allotype is not circulating in Brazil. Unfortunately, the number of samples from Amapá state, which borders French Guiana, was too small to confirm the lack of allotype 13 in Brazil.

Concerning InDel polymorphisms, the CR also varied in the number of tandem nonapeptide repeats (18 to 22), which is a significant factor leading to global isolates’ genetic *pvcsp* diversity. Insertions, resulting in 21 nonapeptide repeats, were mostly detected in BA, while 22 repeats predominated in AF isolates. This number of insertions was similar to those observed in China [63], Cambodia [29], and even in Brazilian Amazon [28]. Interestingly, the deletion of one or two motifs, reducing the number of nonapeptide in tandem to 18 and 19, respectively, was unique detected in BA and bordering countries (Suriname and Venezuela).

Remarkably, samples with deletion showed the most conserved DNA sequence, and, inversely, those with insertion were more polymorphic. Therefore, most allotypes had been arising in the final CR portion (motifs 19 and 20), suggesting more significant selective pressure on these motifs, most likely mediated by the host immune system.

As the sexual recombination during meiosis in the mosquito, CR diversity could be raised by intrahelical strand-slippage events during mitotic DNA replication [28, 70], including inserting or deleting of a repeated motif without changing non-repetitive flanking sequences. However, it is not trivial to discriminate against the influence of these two possibilities unless in low endemic areas, where meiotic recombination rates are far more limited. Thus, the finding of *P*. *vivax* populations with great genomic diversity in several low endemic regions across the world [29, 36, 73], including the AF data here reported for the first time in Brazil, may reinforce the role of mitotic recombination in accelerating CR evolution. However, although human malaria has a very low endemicity in the AF region, parasites are continuously circulating in monkeys/mosquitoes and facing the immune response of this animal host over and over and, consequently, meiotic recombination could also play a role in genetic diversity.

The genetic diversity in South America has been attributed to different parasite lineages originating from geographically diverse regions [12, 36, 73], showing that the demographic history of *P*. *vivax* could also affect the *pvcsp* population structure. Despite the considerable distance between the Brazilian Amazon and Atlantic Forest, the low mobility of infected people between these areas, and the smaller parasite population’s size in AF that generally could reduce gene flow between BA and AF, identical polymorphisms were found in parasite populations of these regions.

The low genetic differentiation between AF and BA populations was also verified with a haplotype network, in which sequences of both regions are interlinked and have similar distances between their haplotypes. AF isolates were formed more clusters than the BA ones. Thus, the isolates of the same geographic region may share similar evolutionary histories. The extensive haplotype distance in BA parasite populations could be a result of i) BA geographic area is much larger than the AF; ii) BA area concentrates approximately 99% of Brazilian *P*. *vivax* cases, increasing the chances of recombination; and iii) BA parasites showed more variation in SNP numbers than AF.

Although AF and BA have distinct epidemiological profiles, including different vector species, transmission rates, and hosts with different immune pressure, migration rates, and regional-temporal fluctuations, we did not detect any fixed nucleotide differences in isolates from these geographic areas. Remarkably, AF autochthonous cases, indicative of zoonotic transmission to humans [6, 10], where parasite populations suffered a bottleneck effect, followed by selective expansion to adapt to non-human host, and different species of anopheline, no systematic genomic changes leading to any consistent signature was recorded.

Contrary to the expectations, all sampled *P*. *vivax* populations harbored high genetic diversity and low level of genetic differentiation between AF and BA parasite populations. These findings were already observed in parasite populations from Colombian geographically distinct regions [74].

The low values of linkage disequilibrium across the *pvcsp* sequence suggest that recombination has not played a crucial role in shuffling the nucleotide variations. Alternatively, the central region of CSP may contain a mutational “hot spot” that could mask the presence of recombination in the *pvcsp* [75].

On the other hand, insertion positions of a small block at the end of the central region with high LD in AF samples could suggest a recent bottleneck and/or clonal expansions, as expected in places with a reduction in transmission rates.

To better understand if selection acts upon the *pvcsp* gene, Tajima’s D neutrality test was performed, and the results suggest that diversity was mediated by balancing selection, probably by the presence of multiple B- and T- cell immunodominant epitopes [19, 20, 48, 49].

It is well known that a single amino acid change or clustered replacements in B- or T- cell epitopes may potentially reduce the antibody response and the peptide-binding pocket of HLA, respectively. Here, we investigated eleven epitopes previously described to trigger the host immune response [20, 33, 48, 49]. Nine of them were in the N- and C- terminal regions, and two in the CR one. The Th2R and Th3R T-cell *P*. *vivax* peptides are located in the C-terminal region and they are orthologous to those of *P*. *falciparum* CSP [76]. The Th2R peptide sequence comprised the P1 T-cytotoxic epitope; polymorphisms in these peptides were present in a small number of Brazilian isolates (13/6%) from AF (4/6%) and BA (9/7%). The Th3R remained conserved in the parasite populations here studied, similar to those of two sub-species of *P*. *ovale* [77], but different to *P*. *malariae* [78], *P*. *knowlesi* [79] and *P*. *falciparum* [76] parasites, in which both Th2R and Th3R epitopes were polymorphic.

To summarize, this study provides information on the genetic polymorphisms of *pvcsp*-gene in isolates from different Brazilian endemic areas, showing high CR diversity and low geographic *pvcsp* population’s structure, probably modulated by natural selection and host immunity. Although the uneven distribution of allelic genes may jeopardize vaccines’ deployment directed to a specific variable *locus*, a unique vaccine formulation could protect human populations in all Brazilian regions.

## Supporting information

S1 Table Number of collected and amplified samples per region/locality. (PDF)

S2 Table Polymorphisms in *pvcsp*-gene fragment observed in Brazilian and foreign country isolates. (PDF)

S3 Table Distribution of allotypes in the CR, and haplotypes. (XLSX)

S1 File Multiple sequence alignment of proteins encoded by *pvcsp-*gene. (PDF)

## List of abbreviations

WHO: World Health Organization
*P. vivax*: *Plasmodium vivax*
CSP: Circumsporozoite protein
*pvcsp*: gene of *Plasmodium vivax* circumsporozoite protein
AF: Atlantic Forest
BA: Brazilian Amazon
NB: Non-Brazilian samples
blast: Basic Local Alignment Search Tool
CR: Central region
Π: Nucleotide diversity
S: polymorphic sites
Hd: Haplotype diversity
Fst: Wright’s fixation statistics
SNP: Single Nucleotide Polymorphism
sSNP: Synonymous Single Nucleotide Polymorphism
nsSNP: Nonsynonymous Single Nucleotide Polymorphism
*An*: *Anopheles*
