# Correction: Balancing selection and high genetic diversity of *Plasmodium vivax* circumsporozoite central region in parasites from Brazilian Amazon and Rio de Janeiro Atlantic Forest

**DOI:** 10.1371/journal.pone.0264054

**Published:** 2022-02-10

**Authors:** Natália Ketrin Almeida-de-Oliveira, Rebecca de Abreu-Fernandes, Lidiane Lima-Cury, Aline Rosa de Lavigne, Anielle de Pina-Costa, Daiana de Souza Perce-da-Silva, Marcos Catanho, Atila Duque Rossi, Patrícia Brasil, Cláudio Tadeu Daniel-Ribeiro, Maria de Fátima Ferreira-da-Cruz

The correct surname for the tenth author, Cláudio Tadeu Daniel-Ribeiro, is Daniel-Ribeiro (not Tadeu Daniel-Ribeiro). The name "Tadeu" is part of the compounding first name (Cláudio Tadeu) and not of the family name.

The author’s name should also be abbreviated as follows: Daniel-Ribeiro, CT.
