# Supplementary material for: Balancing selection and high genetic diversity of Plasmodium vivax circumsporozoite central region in parasites from Brazilian Amazon and Rio de Janeiro Atlantic Forest
Source: PLoS One. 2020 Nov 9;15(11):e0241426. doi: 10.1371/journal.pone.0241426 (PMC7652573; doi:10.1371/journal.pone.0241426)
Supplement: S1 Table — (PDF) [file pone.0241426.s001.pdf]

Table 1: Number of samples collected and amplified per region and sequence information.

Region	n = 239<br>State/Country	n = 219<br>Amplified	SEQUENCE POLYMORPHISMS						
			Central Region						C-terminal
			Motifs number	motif 15	motif 19	motif 20	motif 21	motif 22	G826A / G904A
**Sal-1**	**El Salvador**	**NA**	**20**	**Type 2**	**Type 3**	**Type 3**	**NA**	**NA**	**NA**
BA<br>n=136	Acre (12)	11	20	Type 1<br>Type 2	Type 2<br>Type 3<br>Type 6	Type 2<br>Type 3<br>Type 10<br>Type 11	NP	NP	3 (27%)
	Amapá (9)	8	20 (7)<br>21 (1)	Type 1<br>Type 2	Type 1<br>Type 2<br>Type 3	Type 3<br>Type 10	Type 3	NP	0
	Amazonas (48)	46	18 (3)<br>19 (1)<br>20 (33)<br>21 (6)<br>22 (2)	Type 1<br>Type 2<br>Type 4<br>Type 5	Type 1<br>Type 2<br>Type 3<br>Type 6<br>Type 7	Type 2<br>Type 3<br>Type 10<br>Type 11<br>Type 12	Type 2<br>Type 3<br>Type 10	Type 10	4 (9%)
	Para (55)	52	18 (1)<br>20 (51)	Type 1<br>Type 2<br>Type 4	Type 1<br>Type 2<br>Type 3<br>Type 6<br>Type 7<br>Type 8<br>Type 9	Type 3<br>Type 10	NP	NP	9 (17%)
	Rondônia (17)	17	18 (1)<br>20 (15)<br>22 (1)	Type 1<br>Type 2	Type 1<br>Type 2<br>Type 7	Type 2<br>Type 3<br>Type 10	Type 2	Type 10	0

	Roraima (4)	2	20	Type 1	Type 2	Type 10	NP	NP	0
**AF n=71**	Rio de Janeiro (82)	71	20 (44)<br>21 (1)<br>22 (26)	Type 1<br>Type 2<br>Type 4<br>Type 5	Type 1<br>Type 2<br>Type 3<br>Type 6<br>Type 7<br>Type 8	Type 2<br>Type 3<br>Type 10<br>Type 11<br>Type 12	Type 2<br>Type 10	Type 10	7 (10%)
**NB n=12**	Angola (3)	3	20	Type 1<br>Type 2<br>Type 5	Type 1<br>Type 2<br>Type 9	Type 2<br>Type 3<br>Type 10	NP	NP	0
	Burkina Faso (1)	1	20	Type 1	Type 8	Type 10	NP	NP	0
	Ivory Coast (1)	1	20	Type 2	Type 6	Type 11	NP	NP	0
	Suriname (1)	1	18	Type 1	NP	NP	NP	NP	0
	French Guyana (1)	1	20	Type 1	Type 1	Type 13	NP	NP	0
	Colombia (1)	1	20	Type 2	Type 2	Type 2	NP	NP	0
	Peru (1)	1	20	Type 1	Type 2	Type 3	NP	NP	0
	Venezuela (2)	2	18 (1)<br>20 (1)	Type 1<br>Type 2	Type 2	Type 10	NP	NP	0
	Haiti (1)	1	20	Type 2	Type 2	Type 3	NP	NP	1

Sal-1: reference Salvador 1 strain; BA: Brazilian Amazon; AF: Atlantic Forest; NB: Non-Brazilian; NA: Not Applicable; NP: No Polymorphism; motifs number: nonapeptide in tandem quantity in central region of the gene; Motifs 15, 19, 20, 21 and 22: Nonapeptide positions in central region of the gene and type of nonapeptide designation according to polymorphisms (SNPs).
