# Supplementary material for: Balancing selection and high genetic diversity of Plasmodium vivax circumsporozoite central region in parasites from Brazilian Amazon and Rio de Janeiro Atlantic Forest
Source: PLoS One. 2020 Nov 9;15(11):e0241426. doi: 10.1371/journal.pone.0241426 (PMC7652573; doi:10.1371/journal.pone.0241426)
Supplement: S2 Table — (PDF) [file pone.0241426.s002.pdf]

Polymorphisms in *pvcsp* gene amplified fragment in isolates from Brazilian and Non-Brazilian (NB) regions.

Motif (15)	Nucleotide Position (48)	Codon Position (47)	Isolates				
			AF (71) N (%)	BA (136) N (%)	Total Brazilian (207) N (%)	NB (12) N (%)	Total (219) N (%)
6	T426C	D142D	4 (6%)	22 (16%)	26 (12%)	3 (25%)	29 (13%)
7	C453T	D151D	36 (51%)	65 (48%)	101 (49%)	3 (25%)	104 (47%)
8	A477T	G159G	38 (53%)	35 (26%)	73 (35%)	3 (25%)	76 (35%)
	C480T	D160D	52 (73%)	69 (51%)	121 (58%)	3 (25%)	124 (57%)
	A488C	D163A	26 (37%)	12 (9%)	38 (18%)	1 (8%)	39 (18%)
9	A504T	G168G	49 (69%)	57 (42%)	106 (51%)	5 (42%)	111 (51%)
	A504C	G168G	6 (8%)	7 (5%)	13 (6%)	0	13 (6%)
	C507T	D169D	61 (86%)	80 (59%)	141 (68%)	5 (42%)	146 (67%)
	A515C	D172A	48 (68%)	52 (38%)	100 (48%)	5 (42%)	105 (48%)
	G522A	Q174Q	15 (21%)	52 (38%)	67 (32%)	8 (67%)	75 (34%)
10	T531C	G177G	26 (37%)	17 (13%)	43 (21%)	1 (8%)	44 (20%)
	T531A	G177G	10 (14%)	9 (7%)	19 (9%)	0	19 (9%)
	C542A	A181D	48 (68%)	58 (43%)	106 (51%)	4 (33%)	110 (50%)
	A549G	Q183Q	50 (70%)	60 (44%)	110(53%)	3 (25%)	113 (52%)
11	T558C	G186G	44 (62%)	21 (15%)	65 (31%)	4 (33%)	69 (31%)
	T558A	G186G	5 (7%)	22 (16%)	27 (13%)	0	27 (12%)
	C569A	A190D	40 (56%)	71 (52%)	111 (54%)	5 (42%)	116 (53%)
	G576A	Q192Q	12 (17%)	53 (39%)	65 (31%)	1 (8%)	66 (30%)
12	C585A	G195G	46 (65%)	104 (76%)	150 (72%)	9 (75%)	159 (73%)
	A596C	D199A	24 (34%)	41 (30%)	65 (31%)	8 (67%)	73 (33%)
13	A612T	G204G	9 (13%)	19 (14%)	28 (13%)	3 (25%)	31 (14%)
	A612C	G204G	5 (7%)	12 (9%)	17 (8%)	1 (8%)	18 (8%)
	T615C	D205D	3 (4%)	7 (5%)	10 (5%)	0	10 (5%)
	C623A	A208D	10 (14%)	98 (72%)	108 (52%)	4 (33%)	112 (51%)
14	C639A	G213G	64 (90%)	117 (86%)	181 (87%)	9 (75%)	190 (87%)
	T642C	D214D	4 (6%)	15 (11%)	19 (9%)	1 (8%)	20 (9%)
	A650C	D217A	54 (76%)	37 (27%)	91 (44%)	5 (42%)	96 (44%)
	G657A	Q219Q	44 (62%)	54 (40%)	98 (47%)	4 (33%)	102 (47%)
15	A666T	G222G	42 (59%)	41 (30%)	83 (40%)	4 (33%)	87 (40%)
	T669C	D223D	8 (11%)	4 (3%)	12 (6%)	1 (8%)	13 (6%)
	C677A	A226D	14 (20%)	100 (73%)	114 (55%)	6 (50%)	120 (55%)
	G680T	G227V	6 (8%)	7 (5%)	13 (6%)	1 (8%)	14 (6%)
	A684G	Q228Q	18 (25%)	30 (22%)	48 (23%)	3 (25%)	51 (23%)
16	A693T	G231G	8 (11%)	5 (4%)	13 (6%)	0	13 (6%)
	A704C	D235A	51 (72%)	41 (30%)	92 (44%)	3 (25%)	95 (43%)
	A711G	Q237Q	52 (73%)	83 (61%)	135 (65%)	4 (33%)	139 (63%)
17	A720T	G240G	4 (6%)	43 (32%)	47 (23%)	1 (8%)	48 (22%)
	C731A	A244D	6 (8%)	45 (33%)	51 (25%)	5 (42%)	56 (25%)
18	T750C	D250D	1 (1%)	22 (16%)	23 (11%)	0	23 (10%)

	C758A	A253D	4 (6%)	46 (34%)	50 (24%)	4 (33%)	54 (25%)
	A762T	G254G	2 (3%)	5 (4%)	7 (3%)	3 (25%)	10 (5%)
	G765A	Q255Q	6 (8%)	36 (26%)	42 (20%)	3 (25%)	45 (20%)
**19**	A780T	R260S	4 (6%)	7 (5%)	11 (5%)	2 (17%)	13 (6%)
	C785A	A262D	3 (4%)	25 (18%)	28 (13%)	3 (25%)	31 (14%)
	C785T	A262V	8 (11%)	8 (6%)	16 (8%)	2 (17%)	18 (8%)
	G793C	A265P	67 (94%)	123 (90%)	190 (92%)	10 (83%)	200 (91%)
**20**	G802A	D268N	28 (39%)	97 (71%)	125 (60%)	3 (25%)	128 (58%)
	A807T	R269S	3 (4%)	2 (1%)	5 (2%)	2 (17%)	7 (3%)
	805_07	R269G	28 (39%)	95 (70%)	123 (59%)	3 (25%)	126 (57%)
	C812G	A271G	31 (44%)	89 (65%)	120 (58%)	5 (42%)	125 (57%)
	G820C	A274P	26 (37%)	17 (12%)	43 (21%)	3 (25%)	46 (21%)
**C-terminal**	G826A	G276R	3 (4%)	11 (8%)	14 (7%)	1 (8%)	15 (7%)
	G904A	D302N	4 (6%)	9 (7%)	13 (6%)	0	13 (6%)

The first letter represents the nucleotide in Sal-1 reference sequence and the last the replacing nucleotide; The first letter represents the amino acid in Sal-1 reference sequence and the last the replacing amino acid; AF: Rio de Janeiro Atlantic Forest isolates; Brazilian Amazon isolates; All Brazilian isolates (AF + BA); NB: Non-Brazilian samples from three continents: Africa (5; Burkina Faso, Ivory Coast and 3 from Angola), South America (6; Colombia, French Guyana, Peru, Suriname and 2 from Venezuela) and Central America (1; Haiti); [g]All Brazilian and Non-Brazilian polymorphic isolates.
