# Supplementary material for: Balancing selection and high genetic diversity of Plasmodium vivax circumsporozoite central region in parasites from Brazilian Amazon and Rio de Janeiro Atlantic Forest
Source: PLoS One. 2020 Nov 9;15(11):e0241426. doi: 10.1371/journal.pone.0241426 (PMC7652573; doi:10.1371/journal.pone.0241426)
Supplement: S1 File — (PDF) [file pone.0241426.s004.pdf]

```
                                    10         20         30         40         50         60         70         80         90         100
                           ....|....| ....|....| ....|....| ....|....| ....|....| ....|....| ....|....| ....|....| ....|....| ....|....|
PVX_119355_|_Salvador 1    LLVDLFPTHC GHNVDLSKAI NLNGVNFNNV DASSLGAAHV GQSASRGRGL GENPDDEEGD AKKKKDGKKA EPKNPRENKL KQPGDRADGQ PAGDRADGQP
CSP1_Brazil-BA             .......... .......... .......... .......... .......... .......... .......... .......... .......... ..........
CSP2_Brazil-BA             .......... .......... .......... .......... .......... .......... .......... .......... .......... ..........
CSP3_Brazil-BA             .......... .......... .......... .......... .......... .......... .......... .......... .......... ..........
CSP4_Brazil-BA             .......... .......... .......... .......... .......... .......... .......... .......... .......... ..........
CSP5_Brazil-BA             .......... .......... .......... .......... .......... .......... .......... .......... .......... ..........
CSP6_Brazil-BA             .......... .......... .......... .......... .......... .......... .......... .......... .......... ..........
CSP7_Brazil-BA             .......... .......... .......... .......... .......... .......... .......... .......... .......... ..........
CSP8_Brazil-BA             .......... .......... .......... .......... .......... .......... .......... .......... .......... ..........
CSP9_Brazil-BA             .......... .......... .......... .......... .......... .......... .......... .......... .......... ..........
CSP10_Brazil-BA            .......... .......... .......... .......... .......... .......... .......... .......... .......... ..........
CSP11_Brazil-BA            .......... .......... .......... .......... .......... .......... .......... .......... .......... ..........
CSP12_Brazil-BA            .......... .......... .......... .......... .......... .......... .......... .......... .......... ..........
CSP13_Brazil-BA            .......... .......... .......... .......... .......... .......... .......... .......... .......... ..........
CSP14_Brazil-BA            .......... .......... .......... .......... .......... .......... .......... .......... .......... ..........
CSP15_Brazil-BA            .......... .......... .......... .......... .......... .......... .......... .......... .......... ..........
CSP16_Brazil-BA            .......... .......... .......... .......... .......... .......... .......... .......... .......... ..........
CSP18_Brazil-BA            .......... .......... .......... .......... .......... .......... .......... .......... .......... ..........
CSP19_Brazil-BA            .......... .......... .......... .......... .......... .......... .......... .......... .......... ..........
CSP20_Brazil-BA            .......... .......... .......... .......... .......... .......... .......... .......... .......... ..........
CSP21_Brazil-BA            .......... .......... .......... .......... .......... .......... .......... .......... .......... ..........
CSP22_Brazil-BA            .......... .......... .......... .......... .......... .......... .......... .......... .......... ..........
CSP23_Brazil-BA            .......... .......... .......... .......... .......... .......... .......... .......... .......... ..........
CSP24_Brazil-BA            .......... .......... .......... .......... .......... .......... .......... .......... .......... ..........
CSP25_Brazil-BA            .......... .......... .......... .......... .......... .......... .......... .......... .......... ..........
CSP26_Brazil-BA            .......... .......... .......... .......... .......... .......... .......... .......... .......... ..........
CSP27_Brazil-BA            .......... .......... .......... .......... .......... .......... .......... .......... .......... ..........
CSP28_Brazil-BA            .......... .......... .......... .......... .......... .......... .......... .......... .......... ..........
CSP29_Brazil-BA            .......... .......... .......... .......... .......... .......... .......... .......... .......... ..........
CSP30_Brazil-BA            .......... .......... .......... .......... .......... .......... .......... .......... .......... ..........
CSP31_Brazil-BA            .......... .......... .......... .......... .......... .......... .......... .......... .......... ..........
CSP32_Brazil-BA            .......... .......... .......... .......... .......... .......... .......... .......... .......... ..........
CSP33_Brazil-BA            .......... .......... .......... .......... .......... .......... .......... .......... .......... ..........
CSP34_Brazil-BA            .......... .......... .......... .......... .......... .......... .......... .......... .......... ..........
CSP35_Brazil-BA            .......... .......... .......... .......... .......... .......... .......... .......... .......... ..........
CSP36_Brazil-BA            .......... .......... .......... .......... .......... .......... .......... .......... .......... ..........
CSP37_Brazil-BA            .......... .......... .......... .......... .......... .......... .......... .......... .......... ..........
CSP38_Brazil-BA            .......... .......... .......... .......... .......... .......... .......... .......... .......... ..........
CSP40_Brazil-BA            .......... .......... .......... .......... .......... .......... .......... .......... .......... ..........
CSP41_Brazil-BA            .......... .......... .......... .......... .......... .......... .......... .......... .......... ..........
CSP42_Brazil-BA            .......... .......... .......... .......... .......... .......... .......... .......... .......... ..........
CSP43_Brazil-BA            .......... .......... .......... .......... .......... .......... .......... .......... .......... ..........
CSP44_Brazil-BA            .......... .......... .......... .......... .......... .......... .......... .......... .......... ..........
CSP45_Brazil-BA            .......... .......... .......... .......... .......... .......... .......... .......... .......... ..........
CSP47_Brazil-BA            .......... .......... .......... .......... .......... .......... .......... .......... .......... ..........
CSP48_Brazil-BA            .......... .......... .......... .......... .......... .......... .......... .......... .......... ..........
CSP49_Brazil-BA            .......... .......... .......... .......... .......... .......... .......... .......... .......... ..........
CSP50_Brazil-BA            .......... .......... .......... .......... .......... .......... .......... .......... .......... ..........
CSP52_Brazil-BA            .......... .......... .......... .......... .......... .......... .......... .......... .......... ..........
CSP53_Brazil-BA            .......... .......... .......... .......... .......... .......... .......... .......... .......... ..........
CSP54_Brazil-BA            .......... .......... .......... .......... .......... .......... .......... .......... .......... ..........
CSP55_Brazil-BA            .......... .......... .......... .......... .......... .......... .......... .......... .......... ..........
```

```
CSP56_Brazil-BA            .......... .......... .......... .......... .......... .......... .......... .......... .......... ..........
CSP58_Brazil-BA            .......... .......... .......... .......... .......... .......... .......... .......... .......... ..........
CSP59_Brazil-BA            .......... .......... .......... .......... .......... .......... .......... .......... .......... ..........
                                   10         20         30         40         50         60         70         80         90        100
                           ....|....| ....|....| ....|....| ....|....| ....|....| ....|....| ....|....| ....|....| ....|....| ....|....|
PVX_119355_|_Salvador 1    LLVDLFPTHC GHNVDLSKAI NLNGVNFNNV DASSLGAAHV GQSASRGRGL GENPDDEEGD AKKKKDGKKA EPKNPRENKL KQPGDRADGQ PAGDRADGQP
CSP60_Brazil-BA            .......... .......... .......... .......... .......... .......... .......... .......... .......... ..........
CSP61_Brazil-BA            .......... .......... .......... .......... .......... .......... .......... .......... .......... ..........
CSP62_Brazil-BA            .......... .......... .......... .......... .......... .......... .......... .......... .......... ..........
CSP63_Brazil-BA            .......... .......... .......... .......... .......... .......... .......... .......... .......... ..........
CSP65_Brazil-BA            .......... .......... .......... .......... .......... .......... .......... .......... .......... ..........
CSP66_Brazil-BA            .......... .......... .......... .......... .......... .......... .......... .......... .......... ..........
CSP69_Brazil-BA            .......... .......... .......... .......... .......... .......... .......... .......... .......... ..........
CSP70_Brazil-BA            .......... .......... .......... .......... .......... .......... .......... .......... .......... ..........
CSP71_Brazil-BA            .......... .......... .......... .......... .......... .......... .......... .......... .......... ..........
CSP72_Brazil-BA            .......... .......... .......... .......... .......... .......... .......... .......... .......... ..........
CSP74_Brazil-BA            .......... .......... .......... .......... .......... .......... .......... .......... .......... ..........
CSP75_Brazil-BA            .......... .......... .......... .......... .......... .......... .......... .......... .......... ..........
CSP76_Brazil-BA            .......... .......... .......... .......... .......... .......... .......... .......... .......... ..........
CSP80_Brazil-BA            .......... .......... .......... .......... .......... .......... .......... .......... .......... ..........
CSP82_Brazil-BA            .......... .......... .......... .......... .......... .......... .......... .......... .......... ..........
CSP84_Brazil-BA            .......... .......... .......... .......... .......... .......... .......... .......... .......... ..........
CSP87_Brazil-BA            .......... .......... .......... .......... .......... .......... .......... .......... .......... ..........
CSP88_Brazil-BA            .......... .......... .......... .......... .......... .......... .......... .......... .......... ..........
CSP89_Brazil-BA            .......... .......... .......... .......... .......... .......... .......... .......... .......... ..........
CSP90_Brazil-BA            .......... .......... .......... .......... .......... .......... .......... .......... .......... ..........
CSP91_Brazil-BA            .......... .......... .......... .......... .......... .......... .......... .......... .......... ..........
CSP92_Brazil-BA            .......... .......... .......... .......... .......... .......... .......... .......... .......... ..........
CSP93_Brazil-BA            .......... .......... .......... .......... .......... .......... .......... .......... .......... ..........
CSP95_Brazil-BA            .......... .......... .......... .......... .......... .......... .......... .......... .......... ..........
CSP97_Brazil-BA            .......... .......... .......... .......... .......... .......... .......... .......... .......... ..........
CSP98_Brazil-BA            .......... .......... .......... .......... .......... .......... .......... .......... .......... ..........
CSP101_Brazil-BA           .......... .......... .......... .......... .......... .......... .......... .......... .......... ..........
CSP105_Brazil-BA           .......... .......... .......... .......... .......... .......... .......... .......... .......... ..........
CSP106_Brazil-BA           .......... .......... .......... .......... .......... .......... .......... .......... .......... ..........
CSP107_Brazil-BA           .......... .......... .......... .......... .......... .......... .......... .......... .......... ..........
CSP108_Brazil-BA           .......... .......... .......... .......... .......... .......... .......... .......... .......... ..........
CSP109_Brazil-BA           .......... .......... .......... .......... .......... .......... .......... .......... .......... ..........
CSP110_Brazil-BA           .......... .......... .......... .......... .......... .......... .......... .......... .......... ..........
CSP115_Brazil-BA           .......... .......... .......... .......... .......... .......... .......... .......... .......... ..........
CSP116_Brazil-BA           .......... .......... .......... .......... .......... .......... .......... .......... .......... ..........
CSP118_Brazil-BA           .......... .......... .......... .......... .......... .......... .......... .......... .......... ..........
CSP120_Brazil-BA           .......... .......... .......... .......... .......... .......... .......... .......... .......... ..........
CSP121_Brazil-BA           .......... .......... .......... .......... .......... .......... .......... .......... .......... ..........
CSP122_Brazil-BA           .......... .......... .......... .......... .......... .......... .......... .......... .......... ..........
CSP123_Brazil-BA           .......... .......... .......... .......... .......... .......... .......... .......... .......... ..........
CSP124_Brazil-BA           .......... .......... .......... .......... .......... .......... .......... .......... .......... ..........
CSP125_Brazil-BA           .......... .......... .......... .......... .......... .......... .......... .......... .......... ..........
CSP126_Brazil-BA           .......... .......... .......... .......... .......... .......... .......... .......... .......... ..........
CSP127_Brazil-BA           .......... .......... .......... .......... .......... .......... .......... .......... .......... ..........
CSP128_Brazil-BA           .......... .......... .......... .......... .......... .......... .......... .......... .......... ..........
CSP133_Brazil-BA           .......... .......... .......... .......... .......... .......... .......... .......... .......... ..........
CSP135_Brazil-BA           .......... .......... .......... .......... .......... .......... .......... .......... .......... ..........
CSP142_Brazil-BA           .......... .......... .......... .......... .......... .......... .......... .......... .......... ..........
CSP163_Brazil-BA           .......... .......... .......... .......... .......... .......... .......... .......... .......... ..........
CSP167_Brazil-BA           .......... .......... .......... .......... .......... .......... .......... .......... .......... ..........
CSP170_Brazil-BA           .......... .......... .......... .......... .......... .......... .......... .......... .......... ..........
```

```
CSP174_Brazil-BA         .......... .......... .......... .......... .......... .......... .......... .......... .......... ..........
CSP179_Brazil-BA         .......... .......... .......... .......... .......... .......... .......... .......... .......... ..........
CSP181_Brazil-BA         .......... .......... .......... .......... .......... .......... .......... .......... .......... ..........
                                 10         20         30         40         50         60         70         80         90        100
                         ....|....| ....|....| ....|....| ....|....| ....|....| ....|....| ....|....| ....|....| ....|....| ....|....|
PVX_119355_|_Salvador 1  LLVDLFPTHC GHNVDLSKAI NLNGVNFNNV DASSLGAAHV GQSASRGRGL GENPDDEEGD AKKKKDGKKA EPKNPRENKL KQPGDRADGQ PAGDRADGQP
CSP182_Brazil-BA         .......... .......... .......... .......... .......... .......... .......... .......... .......... ..........
CSP186_Brazil-BA         .......... .......... .......... .......... .......... .......... .......... .......... .......... ..........
CSP190_Brazil-BA         .......... .......... .......... .......... .......... .......... .......... .......... .......... ..........
CSP192_Brazil-BA         .......... .......... .......... .......... .......... .......... .......... .......... .......... ..........
CSP193_Brazil-BA         .......... .......... .......... .......... .......... .......... .......... .......... .......... ..........
CSP194_Brazil-BA         .......... .......... .......... .......... .......... .......... .......... .......... .......... ..........
CSP195_Brazil-BA         .......... .......... .......... .......... .......... .......... .......... .......... .......... ..........
CSP196_Brazil-BA         .......... .......... .......... .......... .......... .......... .......... .......... .......... ..........
CSP197_Brazil-BA         .......... .......... .......... .......... .......... .......... .......... .......... .......... ..........
CSP198_Brazil-BA         .......... .......... .......... .......... .......... .......... .......... .......... .......... ..........
CSP204_Brazil-BA         .......... .......... .......... .......... .......... .......... .......... .......... .......... ..........
CSP210_Brazil-BA         .......... .......... .......... .......... .......... .......... .......... .......... .......... ..........
CSP213_Brazil-BA         .......... .......... .......... .......... .......... .......... .......... .......... .......... ..........
CSP214_Brazil-BA         .......... .......... .......... .......... .......... .......... .......... .......... .......... ..........
CSP222_Brazil-BA         .......... .......... .......... .......... .......... .......... .......... .......... .......... ..........
CSP224_Brazil-BA         .......... .......... .......... .......... .......... .......... .......... .......... .......... ..........
CSP226_Brazil-BA         .......... .......... .......... .......... .......... .......... .......... .......... .......... ..........
CSP227_Brazil-BA         .......... .......... .......... .......... .......... .......... .......... .......... .......... ..........
CSP231_Brazil-BA         .......... .......... .......... .......... .......... .......... .......... .......... .......... ..........
CSP232_Brazil-BA         .......... .......... .......... .......... .......... .......... .......... .......... .......... ..........
CSP235_Brazil-BA         .......... .......... .......... .......... .......... .......... .......... .......... .......... ..........
CSP237_Brazil-BA         .......... .......... .......... .......... .......... .......... .......... .......... .......... ..........
CSP238_Brazil-BA         .......... .......... .......... .......... .......... .......... .......... .......... .......... ..........
CSP239_Brazil-BA         .......... .......... .......... .......... .......... .......... .......... .......... .......... ..........
CSP240_Brazil-BA         .......... .......... .......... .......... .......... .......... .......... .......... .......... ..........
CSP241_Brazil-BA         .......... .......... .......... .......... .......... .......... .......... .......... .......... ..........
CSP245_Brazil-BA         .......... .......... .......... .......... .......... .......... .......... .......... .......... ..........
CSP249_Brazil-BA         .......... .......... .......... .......... .......... .......... .......... .......... .......... ..........
CSP57_Brazil-AF          .......... .......... .......... .......... .......... .......... .......... .......... .......... ..........
CSP64_Brazil-AF          .......... .......... .......... .......... .......... .......... .......... .......... .......... ..........
CSP67_Brazil-AF          .......... .......... .......... .......... .......... .......... .......... .......... .......... ..........
CSP81_Brazil-AF          .......... .......... .......... .......... .......... .......... .......... .......... .......... ..........
CSP86_Brazil-AF          .......... .......... .......... .......... .......... .......... .......... .......... .......... ..........
CSP96_Brazil-AF          .......... .......... .......... .......... .......... .......... .......... .......... .......... ..........
CSP100_Brazil-AF         .......... .......... .......... .......... .......... .......... .......... .......... .......... ..........
CSP102_Brazil-AF         .......... .......... .......... .......... .......... .......... .......... .......... .......... ..........
CSP103_Brazil-AF         .......... .......... .......... .......... .......... .......... .......... .......... .......... ..........
CSP104_Brazil-AF         .......... .......... .......... .......... .......... .......... .......... .......... .......... ..........
CSP130_Brazil-AF         .......... .......... .......... .......... .......... .......... .......... .......... .......... ..........
CSP132_Brazil-AF         .......... .......... .......... .......... .......... .......... .......... .......... .......... ..........
CSP134_Brazil-AF         .......... .......... .......... .......... .......... .......... .......... .......... .......... ..........
CSP136_Brazil-AF         .......... .......... .......... .......... .......... .......... .......... .......... .......... ..........
CSP137_Brazil-AF         .......... .......... .......... .......... .......... .......... .......... .......... .......... ..........
CSP138_Brazil-AF         .......... .......... .......... .......... .......... .......... .......... .......... .......... ..........
CSP139_Brazil-AF         .......... .......... .......... .......... .......... .......... .......... .......... .......... ..........
CSP140_Brazil-AF         .......... .......... .......... .......... .......... .......... .......... .......... .......... ..........
CSP143_Brazil-AF         .......... .......... .......... .......... .......... .......... .......... .......... .......... ..........
CSP144_Brazil-AF         .......... .......... .......... .......... .......... .......... .......... .......... .......... ..........
CSP145_Brazil-AF         .......... .......... .......... .......... .......... .......... .......... .......... .......... ..........
CSP146_Brazil-AF         .......... .......... .......... .......... .......... .......... .......... .......... .......... ..........
CSP147_Brazil-AF         .......... .......... .......... .......... .......... .......... .......... .......... .......... ..........
```

```
CSP148_Brazil-AF              .......... .......... .......... .......... .......... .......... .......... .......... .......... ..........
CSP149_Brazil-AF              .......... .......... .......... .......... .......... .......... .......... .......... .......... ..........
CSP150_Brazil-AF              .......... .......... .......... .......... .......... .......... .......... .......... .......... ..........
                                       10         20         30         40         50         60         70         80         90        100
                              ....|....| ....|....| ....|....| ....|....| ....|....| ....|....| ....|....| ....|....| ....|....| ....|....|
PVX_119355_|_Salvador 1       LLVDLFPTHC GHNVDLSKAI NLNGVNFNNV DASSLGAAHV GQSASRGRGL GENPDDEEGD AKKKKDGKKA EPKNPRENKL KQPGDRADGQ PAGDRADGQP
CSP151_Brazil-AF              .......... .......... .......... .......... .......... .......... .......... .......... .......... ..........
CSP152_Brazil-AF              .......... .......... .......... .......... .......... .......... .......... .......... .......... ..........
CSP153_Brazil-AF              .......... .......... .......... .......... .......... .......... .......... .......... .......... ..........
CSP155_Brazil-AF              .......... .......... .......... .......... .......... .......... .......... .......... .......... ..........
CSP156_Brazil-AF              .......... .......... .......... .......... .......... .......... .......... .......... .......... ..........
CSP159_Brazil-AF              .......... .......... .......... .......... .......... .......... .......... .......... .......... ..........
CSP160_Brazil-AF              .......... .......... .......... .......... .......... .......... .......... .......... .......... ..........
CSP161_Brazil-AF              .......... .......... .......... .......... .......... .......... .......... .......... .......... ..........
CSP162_Brazil-AF              .......... .......... .......... .......... .......... .......... .......... .......... .......... ..........
CSP171_Brazil-AF              .......... .......... .......... .......... .......... .......... .......... .......... .......... ..........
CSP172_Brazil-AF              .......... .......... .......... .......... .......... .......... .......... .......... .......... ..........
CSP173_Brazil-AF              .......... .......... .......... .......... .......... .......... .......... .......... .......... ..........
CSP175_Brazil-AF              .......... .......... .......... .......... .......... .......... .......... .......... .......... ..........
CSP176_Brazil-AF              .......... .......... .......... .......... .......... .......... .......... .......... .......... ..........
CSP177_Brazil-AF              .......... .......... .......... .......... .......... .......... .......... .......... .......... ..........
CSP180_Brazil-AF              .......... .......... .......... .......... .......... .......... .......... .......... .......... ..........
CSP183_Brazil-AF              .......... .......... .......... .......... .......... .......... .......... .......... .......... ..........
CSP184_Brazil-AF              .......... .......... .......... .......... .......... .......... .......... .......... .......... ..........
CSP185_Brazil-AF              .......... .......... .......... .......... .......... .......... .......... .......... .......... ..........
CSP187_Brazil-AF              .......... .......... .......... .......... .......... .......... .......... .......... .......... ..........
CSP188_Brazil-AF              .......... .......... .......... .......... .......... .......... .......... .......... .......... ..........
CSP189_Brazil-AF              .......... .......... .......... .......... .......... .......... .......... .......... .......... ..........
CSP191_Brazil-AF              .......... .......... .......... .......... .......... .......... .......... .......... .......... ..........
CSP200_Brazil-AF              .......... .......... .......... .......... .......... .......... .......... .......... .......... ..........
CSP201_Brazil-AF              .......... .......... .......... .......... .......... .......... .......... .......... .......... ..........
CSP202_Brazil-AF              .......... .......... .......... .......... .......... .......... .......... .......... .......... ..........
CSP203_Brazil-AF              .......... .......... .......... .......... .......... .......... .......... .......... .......... ..........
CSP205_Brazil-AF              .......... .......... .......... .......... .......... .......... .......... .......... .......... ..........
CSP206_Brazil-AF              .......... .......... .......... .......... .......... .......... .......... .......... .......... ..........
CSP207_Brazil-AF              .......... .......... .......... .......... .......... .......... .......... .......... .......... ..........
CSP209_Brazil-AF              .......... .......... .......... .......... .......... .......... .......... .......... .......... ..........
CSP211_Brazil-AF              .......... .......... .......... .......... .......... .......... .......... .......... .......... ..........
CSP212_Brazil-AF              .......... .......... .......... .......... .......... .......... .......... .......... .......... ..........
CSP215_Brazil-AF              .......... .......... .......... .......... .......... .......... .......... .......... .......... ..........
CSP217_Brazil-AF              .......... .......... .......... .......... .......... .......... .......... .......... .......... ..........
CSP218_Brazil-AF              .......... .......... .......... .......... .......... .......... .......... .......... .......... ..........
CSP219_Brazil-AF              .......... .......... .......... .......... .......... .......... .......... .......... .......... ..........
CSP220_Brazil-AF              .......... .......... .......... .......... .......... .......... .......... .......... .......... ..........
CSP221_Brazil-AF              .......... .......... .......... .......... .......... .......... .......... .......... .......... ..........
CSP223_Brazil-AF              .......... .......... .......... .......... .......... .......... .......... .......... .......... ..........
CSP233_Brazil-AF              .......... .......... .......... .......... .......... .......... .......... .......... .......... ..........
CSP234_Brazil-AF              .......... .......... .......... .......... .......... .......... .......... .......... .......... ..........
CSP242_Brazil-AF              .......... .......... .......... .......... .......... .......... .......... .......... .......... ..........
CSP247_Brazil-AF              .......... .......... .......... .......... .......... .......... .......... .......... .......... ..........
CSP250_Brazil-AF              .......... .......... .......... .......... .......... .......... .......... .......... .......... ..........
CSP77_Angola                  .......... .......... .......... .......... .......... .......... .......... .......... .......... ..........
CSP94_Angola                  .......... .......... .......... .......... .......... .......... .......... .......... .......... ..........
CSP119_Angola                 .......... .......... .......... .......... .......... .......... .......... .......... .......... ..........
CSP228_Burkina_Faso           .......... .......... .......... .......... .......... .......... .......... .......... .......... ..........
CSP229_Ivory_Coast            .......... .......... .......... .......... .......... .......... .......... .......... .......... ..........
CSP113_Suriname               .......... .......... .......... .......... .......... .......... .......... .......... .......... ..........
```

```
CSP117_French_Guiana      .......... .......... .......... .......... .......... .......... .......... .......... .......... ..........
CSP129_Haiti              .......... .......... .......... .......... .......... .......... .......... .......... .......... ..........
CSP165_Colombia           .......... .......... .......... .......... .......... .......... .......... .......... .......... ..........
CSP168_Peru               .......... .......... .......... .......... .......... .......... .......... .......... .......... ..........
CSP169_Venezuela          .......... .......... .......... .......... .......... .......... .......... .......... .......... ..........
CSP216_Venezuela          .......... .......... .......... .......... .......... .......... .......... .......... .......... ..........


                                 110        120        130        140        150        160        170        180        190        200
                          ....|....| ....|....| ....|....| ....|....| ....|....| ....|....| ....|....| ....|....| ....|....| ....|....|
PVX_119355_|_Salvador 1   AGDRADGQPA GDRADGQPAG DRAAGQPAGD RADGQPAGDR ADGQPAGDRA DGQPAGDRAD GQPAGDRAAG QPAGDRAAGQ PAGDRADGQP AGDRAAGQPA
CSP1_Brazil-BA            .......... .......... .......... .......... .......... .......... ........D. .......D.. .......... .....D....
CSP2_Brazil-BA            .......... .......... .......... .......... .......... .........A .......... .......D.. .......... .....D....
CSP3_Brazil-BA            .......... .......... .......... .......... .......... .........A .......... .......... .......... .....D....
CSP4_Brazil-BA            .......... .......... .......... .......... .......... A......... .......... .......... .......... .....D....
CSP5_Brazil-BA            .......... .......... .......... .......... .......... .........A .......... .......... ......A... .....D....
CSP6_Brazil-BA            .......... .......... .......... .......... .......... .........A .......... .......D.. ......A... .....D....
CSP7_Brazil-BA            .......... .......... .......... .......... .......... .......... ........D. .......... .......... .....D....
CSP8_Brazil-BA            .......... .......... .......... .......... .......... .......... ........D. .......... .......... .....D....
CSP9_Brazil-BA            .......... .......... .......... .......... .......... .......... ........D. .......... .......... .....D....
CSP10_Brazil-BA           .......... .......... .......... .......... .......... .........A .......... .......... .......... .....D....
CSP11_Brazil-BA           .......... .......... .......... .......... .......... A......... ........D. .......... .......... .....D....
CSP12_Brazil-BA           .......... .......... .......... .......... .......... .......... ........D. .......... ......A... .....D....
CSP13_Brazil-BA           .......... .......... .......... .......... .......... .......... ........D. .......... .......... .....D....
CSP14_Brazil-BA           .......... .......... .......... .......... .......... .......... ........D. .......... .......... .....D....
CSP15_Brazil-BA           .......... .......... .......... .......... .......... .........A .......... .......D.. ......A... .....D....
CSP16_Brazil-BA           .......... .......... .......... .......... .......... .........A .......... .......D.. .......... ..........
CSP18_Brazil-BA           .......... .......... .......... .......... .......... .......... ........D. .......... .......... .....D....
CSP19_Brazil-BA           .......... .......... .......... .......... .......... .......... .......... .......D.. ......A... .....D....
CSP20_Brazil-BA           .......... .......... .......... .......... .......... .......... ........D. .......D.. .......... .....D....
CSP21_Brazil-BA           .......... .......... .......... .......... .......... .......... ........D. .......D.. .......... .....D....
CSP22_Brazil-BA           .......... .......... .......... .......... .......... .......... ........D. .......D.. .......... .....D....
CSP23_Brazil-BA           .......... .......... .......... .......... .......... .......... ........D. .......D.. .......... .....D....
CSP24_Brazil-BA           .......... .......... .......... .......... .......... .........A .......... .......D.. ......A... .....D....
CSP25_Brazil-BA           .......... .......... .......... .......... .......... .......... ........D. .......D.. .......... .....D....
CSP26_Brazil-BA           .......... .......... .......... .......... .......... .......... ........D. .......D.. .......... .....D....
CSP27_Brazil-BA           .......... .......... .......... .......... .......... .......... ........D. .......... .......... .....D....
CSP28_Brazil-BA           .......... .......... .......... .......... .......... .......... ........D. .......D.. .......... .....D....
CSP29_Brazil-BA           .......... .......... .......... .......... .......... .........A .......... .......D.. ......A... .....D....
CSP30_Brazil-BA           .......... .......... .......... .......... .......... .......... ........D. .......D.. .......... .....D....
CSP31_Brazil-BA           .......... .......... .......... .......... .......... .......... ........D. .......D.. .......... ..........
CSP32_Brazil-BA           .......... .......... .......... .......... .......... .........A ........D. .......D.. .......... ..........
CSP33_Brazil-BA           .......... .......... .......... .......... .......... .......... ........D. .......D.. .......... .....D....
CSP34_Brazil-BA           .......... .......... .......... .......... .......... .......... ........D. .......D.. .......... .....D....
CSP35_Brazil-BA           .......... .......... .......... .......... .......... .......... ........D. .......D.. .......... .....D....
CSP36_Brazil-BA           .......... .......... .......... .......... .......... A......... ........D. .......... .......... .....D....
CSP37_Brazil-BA           .......... .......... .......... .......... .......... .......... ........D. .......D.. .......... .....D....
CSP38_Brazil-BA           .......... .......... .......... .......... .......... A........A ........D. .......... .......... .....D....
CSP40_Brazil-BA           .......... .......... .......... .......... .......... .......... ........D. .......... ......A... .....D....
CSP41_Brazil-BA           .......... .......... .......... .......... .......... .......... ........D. .......D.. .......... .....D....
CSP42_Brazil-BA           .......... .......... .......... .......... .......... .........A ........D. .......D.. .......... ..........
CSP43_Brazil-BA           .......... .......... .......... .......... .......... A......... ........D. .......D.. .......... .....D....
CSP44_Brazil-BA           .......... .......... .......... .......... .......... .......... ........D. .......D.. .......... .....D....
```

```
CSP45_Brazil-BA       .......... .......... .......... .......... .......... .......... ........D. .......D.. .......... .....D....
CSP47_Brazil-BA       .......... .......... .......... .......... .......... A........A .......... .......D.. ......A... .....D....
CSP48_Brazil-BA       .......... .......... .......... .......... .......... .......... ........D. .......D.. ......A... ..........
                               110        120        130        140        150        160        170        180        190        200
                      ....|....| ....|....| ....|....| ....|....| ....|....| ....|....| ....|....| ....|....| ....|....| ....|....|
PVX_119355_|_Salvador 1 AGDRADGQPA GDRADGQPAG DRAAGQPAGD RADGQPAGDR ADGQPAGDRA DGQPAGDRAD GQPAGDRAAG QPAGDRAAGQ PAGDRADGQP AGDRAAGQPA
CSP49_Brazil-BA       .......... .......... .......... .......... .......... .........A ........D. .......D.. ......A... .....D....
CSP50_Brazil-BA       .......... .......... .......... .......... .......... .........A .......... .......D.. ......A... .....D....
CSP52_Brazil-BA       .......... .......... .......... .......... .......... .........A .......... .......D.. ......A... .....D....
CSP53_Brazil-BA       .......... .......... .......... .......... .......... .......... ........D. .......D.. .......... .....D....
CSP54_Brazil-BA       .......... .......... .......... .......... .......... .........A .......... .......D.. ......A... .....D....
CSP55_Brazil-BA       .......... .......... .......... .......... .......... .........A .......... .......... .......... ..........
CSP56_Brazil-BA       .......... .......... .......... .......... .......... .......... ........D. .......D.. .......... .....D....
CSP58_Brazil-BA       .......... .......... .......... .......... .......... .........A .......... .......D.. ......A... .....D....
CSP59_Brazil-BA       .......... .......... .......... .......... .......... .......... .......... .......... .......... ..........
CSP60_Brazil-BA       .......... .......... .......... .......... .......... .........A .......... .......... ......A... .....D....
CSP61_Brazil-BA       .......... .......... .......... .......... .......... .......... ........D. .......... .......... .....D....
CSP62_Brazil-BA       .......... .......... .......... .......... .......... .......... .......... .......... .......... ..........
CSP63_Brazil-BA       .......... .......... .......... .......... .......... .......... ........D. .......D.. .......... .....D....
CSP65_Brazil-BA       .......... .......... .......... .......... .......... .......... .......... .......... .......... ..........
CSP66_Brazil-BA       .......... .......... .......... .......... .......... .......... .......... .......... .......... ..........
CSP69_Brazil-BA       .......... .......... .......... .......... .......... .........A ........D. .......D.. ......A... .....D....
CSP70_Brazil-BA       .......... .......... .......... .......... .......... .........A .......... .......D.. .......... .....D....
CSP71_Brazil-BA       .......... .......... .......... .......... .......... .......... ........D. .......... .......... .....D....
CSP72_Brazil-BA       .......... .......... .......... .......... .......... A........A .......... .......D.. .......... .....D....
CSP74_Brazil-BA       .......... .......... .......... .......... .......... .......... .......... .......... .......... .....D....
CSP75_Brazil-BA       .......... .......... .......... .......... .......... .......... .......... .......... .......... ..........
CSP76_Brazil-BA       .......... .......... .......... .......... .......... .......... .......... .......... .......... .....D....
CSP80_Brazil-BA       .......... .......... .......... .......... .......... .........A ........D. .......D.. .......... .....D....
CSP82_Brazil-BA       .......... .......... .......... .......... .......... .......... .......... .......... .......... .....D....
CSP84_Brazil-BA       .......... .......... .......... .......... .......... A........A .......... .......... .......... .....D....
CSP87_Brazil-BA       .......... .......... .......... .......... .......... .......... .......... .......... .......... .....D....
CSP88_Brazil-BA       .......... .......... .......... .......... .......... .......... .......... .......... .......... .....D....
CSP89_Brazil-BA       .......... .......... .......... .......... .......... .........A .......... .......D.. ......A... .....D....
CSP90_Brazil-BA       .......... .......... .......... .......... .......... .......... .......... .......... .......... .....D....
CSP91_Brazil-BA       .......... .......... .......... .......... .......... .......... .......... .......... .......... ..........
CSP92_Brazil-BA       .......... .......... .......... .......... .......... A........A ........D. .......D.. .......... ..........
CSP93_Brazil-BA       .......... .......... .......... .......... .......... .......... ........D. .......D.. .......... .....D....
CSP95_Brazil-BA       .......... .......... .......... .......... .......... .......... .......... .......... .......... ..........
CSP97_Brazil-BA       .......... .......... .......... .......... .......... .......... ........D. .......D.. .......... .....D....
CSP98_Brazil-BA       .......... .......... .......... .......... .......... .........A .......... .......D.. ......A... .....D....
CSP101_Brazil-BA      .......... .......... .......... .......... .......... .........A .......... .......D.. ......A... .....D....
CSP105_Brazil-BA      .......... .......... .......... .......... .......... .......... .......... .......... .......... ..........
CSP106_Brazil-BA      .......... .......... .......... .......... .......... .......... ........D. .......D.. .......... .....D....
CSP107_Brazil-BA      .......... .......... .......... .......... .......... .......... .......... .......... .......... .....D....
CSP108_Brazil-BA      .......... .......... .......... .......... .......... .......... ........D. .......D.. .......... .....D....
CSP109_Brazil-BA      .......... .......... .......... .......... .......... A........A ........D. .......... .......... ..........
CSP110_Brazil-BA      .......... .......... .......... .......... .......... .......... .......... .......... .......... .....D....
CSP115_Brazil-BA      .......... .......... .......... .......... .......... .........A ........D. .......D.. ......A... .....D....
CSP116_Brazil-BA      .......... .......... .......... .......... .......... .......... .......... .......... .......... ..........
CSP118_Brazil-BA      .......... .......... .......... .......... .......... .......... ........D. .......D.. .......... .....D....
CSP120_Brazil-BA      .......... .......... .......... .......... .......... .........A .......... .......D.. ......A... .....D....
CSP121_Brazil-BA      .......... .......... .......... .......... .......... .........A .......... .......D.. ......A... .....D....
CSP122_Brazil-BA      .......... .......... .......... .......... .......... .........A .......... .......D.. ......A... .....D....
CSP123_Brazil-BA      .......... .......... .......... .......... .......... .......... .......... .......... .......... ..........
CSP124_Brazil-BA      .......... .......... .......... .......... .......... .......... .......... .......... .......... .....D....
CSP125_Brazil-BA      .......... .......... .......... .......... .......... .......... ........D. .......D.. .......... .....D....
```

```
CSP126_Brazil-BA      .......... .......... .......... .......... .......... .......... ........D. .......D.. .......... .....D....
CSP127_Brazil-BA      .......... .......... .......... .......... .......... .......... .......... .......... .......... ..........
CSP128_Brazil-BA      .......... .......... .......... .......... .......... .......... .......... .......... .......... ..........
                              110        120        130        140        150        160        170        180        190        200
                      ....|....| ....|....| ....|....| ....|....| ....|....| ....|....| ....|....| ....|....| ....|....| ....|....|
PVX_119355_|_Salvador 1 AGDRADGQPA GDRADGQPAG DRAAGQPAGD RADGQPAGDR ADGQPAGDRA DGQPAGDRAD GQPAGDRAAG QPAGDRAAGQ PAGDRADGQP AGDRAAGQPA
CSP133_Brazil-BA      .......... .......... .......... .......... .......... .......... .......... .......... .......... ..........
CSP135_Brazil-BA      .......... .......... .......... .......... .......... .......... ........D. .......D.. .......... ..........
CSP142_Brazil-BA      .......... .......... .......... .......... .......... .........A .......... .......... ......A... ..........
CSP163_Brazil-BA      .......... .......... .......... .......... .......... .......... ........D. .......D.. ......A... ..........
CSP167_Brazil-BA      .......... .......... .......... .......... .......... .......... .......... .......... .......... ..........
CSP170_Brazil-BA      .......... .......... .......... .......... .......... .......... .......... .......... ......A... ..........
CSP174_Brazil-BA      .......... .......... .......... .......... .......... .......... .......... .......... .......... ..........
CSP179_Brazil-BA      .......... .......... .......... .......... .......... .........A .......... .......D.. ......A... .....D....
CSP181_Brazil-BA      .......... .......... .......... .......... .......... .........A .......... .......D.. ......A... .....D....
CSP182_Brazil-BA      .......... .......... .......... .......... .......... .........A .......... .......D.. ......A... .....D....
CSP186_Brazil-BA      .......... .......... .......... .......... .......... .......... .......... .......... .......... ..........
CSP190_Brazil-BA      .......... .......... .......... .......... .......... .......... .......... .......... .......... ..........
CSP192_Brazil-BA      .......... .......... .......... .......... .......... A........A ........D. .......D.. .......... ..........
CSP193_Brazil-BA      .......... .......... .......... .......... .......... .........A .......... .......D.. .......... .....D....
CSP194_Brazil-BA      .......... .......... .......... .......... .......... .........A .......... .......D.. ......A... .....D....
CSP195_Brazil-BA      .......... .......... .......... .......... .......... A........A .......... .......... .......... ..........
CSP196_Brazil-BA      .......... .......... .......... .......... .......... .......... .......... .......... .......... ..........
CSP197_Brazil-BA      .......... .......... .......... .......... .......... .......... ........D. .......D.. .......... .....D....
CSP198_Brazil-BA      .......... .......... .......... .......... .......... .......... .......... .......... .......... ..........
CSP204_Brazil-BA      .......... .......... .......... .......... .......... .......... .......... .......... .......... ..........
CSP210_Brazil-BA      .......... .......... .......... .......... .......... .........A .......... .......D.. ......A... .....D....
CSP213_Brazil-BA      .......... .......... .......... .......... .......... .......... ........D. .......D.. .......... .....D....
CSP214_Brazil-BA      .......... .......... .......... .......... .......... .......... ........D. .......... .......... .....D....
CSP222_Brazil-BA      .......... .......... .......... .......... .......... .......... .......... .......... .......... ..........
CSP224_Brazil-BA      .......... .......... .......... .......... .......... .........A .......... .......D.. ......A... .....D....
CSP226_Brazil-BA      .......... .......... .......... .......... .......... .........A .......... .......D.. ......A... .....D....
CSP227_Brazil-BA      .......... .......... .......... .......... .......... .........A .......... .......D.. .......... .....D....
CSP231_Brazil-BA      .......... .......... .......... .......... .......... .........A .......... .......... ......A... .....D....
CSP232_Brazil-BA      .......... .......... .......... .......... .......... .........A .......... .......... ......A... .....D....
CSP235_Brazil-BA      .......... .......... .......... .......... .......... .........A .......... .......... ......A... .....D....
CSP237_Brazil-BA      .......... .......... .......... .......... .......... .......... .......... .......... ......A... .....D....
CSP238_Brazil-BA      .......... .......... .......... .......... .......... .........A .......... .......D.. ......A... .....D....
CSP239_Brazil-BA      .......... .......... .......... .......... .......... .......... ........D. .......... .......... .....D....
CSP240_Brazil-BA      .......... .......... .......... .......... .......... .......... .......... .......... .......... ..........
CSP241_Brazil-BA      .......... .......... .......... .......... .......... .......... .......... .......... .......... .....D....
CSP245_Brazil-BA      .......... .......... .......... .......... .......... .........A .......... .......... ......A... ..........
CSP249_Brazil-BA      .......... .......... .......... .......... .......... .........A .......... .......... ......A... ..........
CSP57_Brazil-AF       .......... .......... .......... .......... .......... .........A .......... .......... ......A... .....D....
CSP64_Brazil-AF       .......... .......... .......... .......... .......... .......... .......... .......... ......A... ..........
CSP67_Brazil-AF       .......... .......... .......... .......... .......... .......... .......... .......D.. .......... ..........
CSP81_Brazil-AF       .......... .......... .......... .......... .......... .......... .......... .......... ......A... ..........
CSP86_Brazil-AF       .......... .......... .......... .......... .......... .........A ........D. .......D.. ......A... .....D....
CSP96_Brazil-AF       .......... .......... .......... .......... .......... A........A ........D. .......D.. .......... .....D....
CSP100_Brazil-AF      .......... .......... .......... .......... .......... .........A .......... .......D.. .......... ..........
CSP102_Brazil-AF      .......... .......... .......... .......... .......... .........A .......... .......D.. .......... ..........
CSP103_Brazil-AF      .......... .......... .......... .......... .......... .........A .......... .......... .......... ..........
CSP104_Brazil-AF      .......... .......... .......... .......... .......... A........A ........D. .......D.. .......... ..........
CSP130_Brazil-AF      .......... .......... .......... .......... .......... .......... ........D. .......... ......A... ..........
CSP132_Brazil-AF      .......... .......... .......... .......... .......... A........A ........D. .......D.. .......... ..........
CSP134_Brazil-AF      .......... .......... .......... .......... .......... .......... ........D. .......... .......... ..........
CSP136_Brazil-AF      .......... .......... .......... .......... .......... A........A ........D. .......D.. .......... ..........
```

```
CSP137_Brazil-AF       .......... .......... .......... .......... .......... .......... ........D. .......D.. ......A... ..........
CSP138_Brazil-AF       .......... .......... .......... .......... .......... A........A ........D. .......D.. .......... ..........
CSP139_Brazil-AF       .......... .......... .......... .......... .......... .........A ........D. .......... ......A... ..........
                              110        120        130        140        150        160        170        180        190        200
                       ....|....| ....|....| ....|....| ....|....| ....|....| ....|....| ....|....| ....|....| ....|....| ....|....|
PVX_119355_|_Salvador 1 AGDRADGQPA GDRADGQPAG DRAAGQPAGD RADGQPAGDR ADGQPAGDRA DGQPAGDRAD GQPAGDRAAG QPAGDRAAGQ PAGDRADGQP AGDRAAGQPA
CSP140_Brazil-AF       .......... .......... .......... .......... .......... A........A ........D. .......D.. .......... ..........
CSP143_Brazil-AF       .......... .......... .......... .......... .......... A........A ........D. .......D.. .......... ..........
CSP144_Brazil-AF       .......... .......... .......... .......... .......... .......... ........D. .......D.. ......A... ..........
CSP145_Brazil-AF       .......... .......... .......... .......... .......... A........A ........D. .......D.. .......... ..........
CSP146_Brazil-AF       .......... .......... .......... .......... .......... A........A ........D. .......D.. .......... ..........
CSP147_Brazil-AF       .......... .......... .......... .......... .......... A........A ........D. .......D.. .......... ..........
CSP148_Brazil-AF       .......... .......... .......... .......... .......... A........A ........D. .......D.. .......... ..........
CSP149_Brazil-AF       .......... .......... .......... .......... .......... A......... ........D. .......D.. .......... ..........
CSP150_Brazil-AF       .......... .......... .......... .......... .......... .......... .......... .......... ......A... ..........
CSP151_Brazil-AF       .......... .......... .......... .......... .......... A........A ........D. .......D.. .......... ..........
CSP152_Brazil-AF       .......... .......... .......... .......... .......... .......... ........D. .......... ......A... ..........
CSP153_Brazil-AF       .......... .......... .......... .......... .......... .........A ........D. .......D.. .......... ..........
CSP155_Brazil-AF       .......... .......... .......... .......... .......... A......... .......... .......D.. .......... ..........
CSP156_Brazil-AF       .......... .......... .......... .......... .......... .......... ........D. .......... .......... ..........
CSP159_Brazil-AF       .......... .......... .......... .......... .......... A........A ........D. .......D.. .......... ..........
CSP160_Brazil-AF       .......... .......... .......... .......... .......... .........A ........D. .......D.. .......... ..........
CSP161_Brazil-AF       .......... .......... .......... .......... .......... .........A .......... .......D.. ......A... ..........
CSP162_Brazil-AF       .......... .......... .......... .......... .......... A........A ........D. .......D.. ......A... ..........
CSP171_Brazil-AF       .......... .......... .......... .......... .......... A........A ........D. .......D.. .......... ..........
CSP172_Brazil-AF       .......... .......... .......... .......... .......... .......... .......... .......... .......... ..........
CSP173_Brazil-AF       .......... .......... .......... .......... .......... .......... ........D. .......... ......A... ..........
CSP175_Brazil-AF       .......... .......... .......... .......... .......... A........A .......... .......... .......... ..........
CSP176_Brazil-AF       .......... .......... .......... .......... .......... A........A .......... .......... .......... ..........
CSP177_Brazil-AF       .......... .......... .......... .......... .......... A........A .......... .......... .......... ..........
CSP180_Brazil-AF       .......... .......... .......... .......... .......... .........A .......... .......D.. .......... ..........
CSP183_Brazil-AF       .......... .......... .......... .......... .......... A........A ........D. .......D.. .......... ..........
CSP184_Brazil-AF       .......... .......... .......... .......... .......... .......... .......... .......D.. ......A... .....D....
CSP185_Brazil-AF       .......... .......... .......... .......... .......... A........A ........D. .......D.. .......... ..........
CSP187_Brazil-AF       .......... .......... .......... .......... .......... A......... ........D. .......... .......... ..........
CSP188_Brazil-AF       .......... .......... .......... .......... .......... .........A ........D. .......... .......... ..........
CSP189_Brazil-AF       .......... .......... .......... .......... .......... A........A ........D. .......D.. .......... ..........
CSP191_Brazil-AF       .......... .......... .......... .......... .......... .........A .......... .......D.. ......A... .....D....
CSP200_Brazil-AF       .......... .......... .......... .......... .......... .........A .......... .......D.. .......... ..........
CSP201_Brazil-AF       .......... .......... .......... .......... .......... .......... ........D. .......... ......A... ..........
CSP202_Brazil-AF       .......... .......... .......... .......... .......... .........A ........D. .......... ......A... ..........
CSP203_Brazil-AF       .......... .......... .......... .......... .......... .........A ........D. .......... ......A... ..........
CSP205_Brazil-AF       .......... .......... .......... .......... .......... .......... ........D. .......D.. .......... ..........
CSP206_Brazil-AF       .......... .......... .......... .......... .......... .........A .......... .......D.. .......... ..........
CSP207_Brazil-AF       .......... .......... .......... .......... .......... .........A ........D. .......D.. .......... ..........
CSP209_Brazil-AF       .......... .......... .......... .......... .......... .......... ........D. .......D.. .......... .....D....
CSP211_Brazil-AF       .......... .......... .......... .......... .......... .........A ........D. .......D.. .......... .....D....
CSP212_Brazil-AF       .......... .......... .......... .......... .......... A........A .......... .......D.. ......A... ..........
CSP215_Brazil-AF       .......... .......... .......... .......... .......... .........A .......... .......... .......... ..........
CSP217_Brazil-AF       .......... .......... .......... .......... .......... .......... ........D. .......... ......A... ..........
CSP218_Brazil-AF       .......... .......... .......... .......... .......... A........A ........D. .......... ......A... ..........
CSP219_Brazil-AF       .......... .......... .......... .......... .......... .......... ........D. .......D.. ......A... ..........
CSP220_Brazil-AF       .......... .......... .......... .......... .......... .........A ........D. .......... ......A... ..........
CSP221_Brazil-AF       .......... .......... .......... .......... .......... .........A ........D. .......... .......... ..........
CSP223_Brazil-AF       .......... .......... .......... .......... .......... .......... .......... .......... .......... .....D....
CSP233_Brazil-AF       .......... .......... .......... .......... .......... .........A ........D. .......... .......... .....D....
CSP234_Brazil-AF       .......... .......... .......... .......... .......... .........A ........D. .......... ......A... ..........
```

```
CSP242_Brazil-AF          .......... .......... .......... .......... .......... .........A .......... .......... .......... .....D....
CSP247_Brazil-AF          .......... .......... .......... .......... .......... .......... ........D. .......... .......... ..........
CSP250_Brazil-AF          .......... .......... .......... .......... .......... .........A ........D. .......... .......... ..........
                                 110        120        130        140        150        160        170        180        190        200
                          ....|....| ....|....| ....|....| ....|....| ....|....| ....|....| ....|....| ....|....| ....|....| ....|....|
PVX_119355_|_Salvador 1   AGDRADGQPA GDRADGQPAG DRAAGQPAGD RADGQPAGDR ADGQPAGDRA DGQPAGDRAD GQPAGDRAAG QPAGDRAAGQ PAGDRADGQP AGDRAAGQPA
CSP77_Angola              .......... .......... .......... .......... .......... .......... .......... .......... .....A.... ..........
CSP94_Angola              .......... .......... .......... .......... .......... .......... ........D. .......D.. .......... .....D....
CSP119_Angola             .......... .......... .......... .......... .......... .........A ........D. .......... .....A.... ..........
CSP228_Burkina_Faso       .......... .......... .......... .......... .......... .........A ........D. .......D.. .....A.... ..........
CSP229_Ivory_Coast        .......... .......... .......... .......... .......... A........A .......... .......... .....A.... ..........
CSP113_Suriname           .......... .......... .......... .......... .......... .......... ........D. .......D.. .......... .....D....
CSP117_French_Guiana      .......... .......... .......... .......... .......... .......... .......... .......... .....A.... .....D....
CSP129_Haiti              .......... .......... .......... .......... .......... .........A .......... .......D.. .....A.... ..........
CSP165_Colombia           .......... .......... .......... .......... .......... .......... .......... .......... .....A.... ..........
CSP168_Peru               .......... .......... .......... .......... .......... .......... .......... .......... .......... ..........
CSP169_Venezuela          .......... .......... .......... .......... .......... .......... .......... .......... .......... ..........
CSP216_Venezuela          .......... .......... .......... .......... .......... .........A .......... .......D.. .....A.... .....D....

                                 210        220        230        240        250        260        270        280        290        300
                          ....|....| ....|....| ....|....| ....|....| ....|....| ....|....| ....|....| ....|....| ....|....| ....|....|
PVX_119355_|_Salvador 1   GDRADGQPAG DRAAGQPAGD RADGQPAGDR AAGQPAGDRA AGQPAGDRAA GQAAGDRAAG QAA------- ---------- -GGNAGGQGQ NNEGANAPNE
CSP1_Brazil-BA            .......... ...D...... ..A....... .......... D........V ..P..NG.G. ...------- ---------- -......... ..........
CSP2_Brazil-BA            .......... ...DV..... .......... .......... .......... ..P..NG.G. ...------- ---------- -R........ ..........
CSP3_Brazil-BA            .......... ...D...... .......... .......... .......... ..P..NG.G. ...------- ---------- -R........ ..........
CSP4_Brazil-BA            .......... ...D...... .......... .......... .......... ..P..NG.G. ...------- ---------- -......... ..........
CSP5_Brazil-BA            .......... ...D...... ..A....... .......... .......... ..P..NG.G. ...------- ---------- -......... ..........
CSP6_Brazil-BA            ....A..... ...D...... ..A....... .......... .......... ..P..NG.G. ...------- ---------- -......... ..........
CSP7_Brazil-BA            .......... ...D...... .......... .D........ D........D ..P..NG.G. ...------- ---------- -......... ..........
CSP8_Brazil-BA            .......... ...D...... .......... .D........ .........D ..P..NG.G. ...------- ---------- -......... ..........
CSP9_Brazil-BA            .......... ...D...... .......... .D........ D......... ..P..NG.G. ...------- ---------- -......... ..........
CSP10_Brazil-BA           .......... ...DV..... .......... .D........ D........D ..P..NG.G. ...------- ---------- -R........ ..........
CSP11_Brazil-BA           .......... ...D...... .......... .D........ D......... ..P..NG.G. ...------- ---------- -......... ..........
CSP12_Brazil-BA           .......... .......... .......... .......... D......... ..P..NG.G. ...------- ---------- -......... ..........
CSP13_Brazil-BA           .......... ...D...... .......... .......... .........D ..P..NG.G. ...------- ---------- -......... ..........
CSP14_Brazil-BA           .......... ...D...... .......... .D........ D........D ..P..NG.G. ...------- ---------- -......... ..........
CSP15_Brazil-BA           ....A..... ...D...... ..A....... .......... .......... ..P..NG.G. ...------- ---------- -......... ..........
CSP16_Brazil-BA           ....A..... .......... ..A....... .......... .......... ..P..NG.G. ...------- ---------- -R........ ..........
CSP18_Brazil-BA           .......... ...D...... .......... .D........ D......... ..P..NG.G. ...------- ---------- -......... ..........
CSP19_Brazil-BA           .......... ...D...... ..A....... .......... .......... ..P..NG.G. ...------- ---------- -......... ..........
CSP20_Brazil-BA           .......... ...D...... .......... .......... .......... ..P..NG.G. ...------- ---------- -......... ..........
CSP21_Brazil-BA           .......... ...D...... .......... .D........ D......... ..P..NG.G. ...------- ---------- -R........ ..........
CSP22_Brazil-BA           .......... ...D...... .......... .D........ D......... ..P..NG.G. ...------- ---------- -......... ..........
CSP23_Brazil-BA           .......... ...D...... .......... .D........ D......... ..P..NG.G. ...------- ---------- -......... ..........
CSP24_Brazil-BA           ....A..... ...D...... ..A....... .......... .......... ..P..NG.G. ...------- ---------- -......... ..........
CSP25_Brazil-BA           .......... ...D...... .......... .D........ D........D ..P..NG.G. ...------- ---------- -......... ..........
CSP26_Brazil-BA           .......... ...D...... .......... .D........ D........D ..P..NG.G. ...------- ---------- -......... ..........
CSP27_Brazil-BA           .......... ...D...... .......... .D........ D........D ..P..NG.G. ...------- ---------- -......... ..........
CSP28_Brazil-BA           .......... .......... .......... .......... .......... .....NG.G. ...------- ---------- -......... ..........
CSP29_Brazil-BA           ....A..... ...D...... ..A....... .......... .......... ..P..NG.G. ...------- ---------- -......... ..........
CSP30_Brazil-BA           .......... ...D...... .......... .......... .........V ..P..NG.G. ...------- ---------- -......... ..........
CSP31_Brazil-BA           .......... ...DV..... .......... .D........ D......S.D ..P..NG.G. ...------- ---------- -......... ..........
CSP32_Brazil-BA           .......... ...D...... .......... .D........ D........D ..P..NG.G. ...------- ---------- -......... ..........
CSP33_Brazil-BA           .......... ...D...... .......... .D........ D........D ..P..NG.G. ...------- ---------- -......... ..........
CSP34_Brazil-BA           .......... ...D...... .......... .D........ D......... ..P..NG.G. ...------- ---------- -......... ..........
CSP35_Brazil-BA           .......... ...D...... .......... .......... .......... ..P..NG.G. ...------- ---------- -......... ..........
CSP36_Brazil-BA           .......... ...D...... .......... .D........ D........D ..P..NG.G. ...------- ---------- -......... ..........
```

```
CSP37_Brazil-BA     .......... ...D...... .......... .......... D......... ..P..NG.G. ...------- ---------- -......... ..........
CSP38_Brazil-BA     .......... ...D...... .......... .D........ D......... ..P..NG.G. ...------- ---------- -......... ..........
CSP40_Brazil-BA     .......... .......... .......... .D........ .......... ..P..NG.G. ...------- ---------- -......... ..........
                           210        220        230        240        250        260        270        280        290        300
                    ....|....| ....|....| ....|....| ....|....| ....|....| ....|....| ....|....| ....|....| ....|....| ....|....|
PVX_119355_|_Salvador 1  GDRADGQPAG DRAAGQPAGD RADGQPAGDR AAGQPAGDRA AGQPAGDRAA GQAAGDRAAG QAA------- ---------- -GGNAGGQGQ NNEGANAPNE
CSP41_Brazil-BA     .......... ...D...... .......... .D........ D......... .....NG.G. ...------- ---------- -......... ..........
CSP42_Brazil-BA     ....A..... ...DV..... ..A....... .D........ D......S.. ..P..NG.G. ...------- ---------- -......... ..........
CSP43_Brazil-BA     .......... ...D...... ..A....... .D........ D........D ..P..NG.G. ...------- ---------- -......... ..........
CSP44_Brazil-BA     .......... ...D...... .......... .......... .......... ..P..NG.G. ...------- ---------- -......... ..........
CSP45_Brazil-BA     .......... ...D...... .......... .D........ D......... ..P..NG.G. ...------- ---------- -......... ..........
CSP47_Brazil-BA     .......... ...DV..... .......... .......... D........V ..P..NG.G. ...------- ---------- -......... ..........
CSP48_Brazil-BA     .......... .......... .......... .......... D......... ..P..NG.G. ...------- ---------- -......... ..........
CSP49_Brazil-BA     .......... ...D...... .......... .......... .......... ..P..NG.G. ...------- ---------- -......... ..........
CSP50_Brazil-BA     ....A..... ...D...... .......... .......... .......S.V ..P..NG.G. ...------- ---------- -......... ..........
CSP52_Brazil-BA     ....A..... ...D...... ..A....... .......... .......... ..P..NG.G. ...------- ---------- -......... ..........
CSP53_Brazil-BA     .......... ...D...... .......... .......... D....----- ---------- ---------- ---------- -......... ..........
CSP54_Brazil-BA     ....A..... ...D...... ..A....... .......... .......... ..P..NG.G. ...------- ---------- -......... ..........
CSP55_Brazil-BA     .......... .......... .......... .......... .......... ..P..NG.G. ...------- ---------- -......... ..........
CSP56_Brazil-BA     .......... ...D...... .......... .D........ D........D ..P..NG.G. ...------- ---------- -......... ..........
CSP58_Brazil-BA     ....A..... ...D...... ..A....... .......... .......... ..P..NG.G. ...------- ---------- -......... ..........
CSP59_Brazil-BA     .......... .......... .......... .......... .......... ..P..N.... .P.------- ---------- -......... ..........
CSP60_Brazil-BA     ....A..... ...D...... ..A....... .......... .......... ..P..NG.G. ...------- ---------- -......... ..........
CSP61_Brazil-BA     .......... ...D...... .......... .D........ D........D ..P..NG.G. ...------- ---------- -......... ..........
CSP62_Brazil-BA     .......... .......... ..A....... .......... .......... .......... ...------- ---------- R......... ..........
CSP63_Brazil-BA     .......... ...D...... .......... .D........ .....----- ---------- ---------- ---------- -......... ..........
CSP65_Brazil-BA     .......... ...D...... .......... .......... .......... ..P....... ...------- ---------- -......... ..........
CSP66_Brazil-BA     .......... .......... .......... .......... .......... ..P....... ...------- ---------- -......... ..........
CSP69_Brazil-BA     ....A..... ...D...... .......... .......... .......... ..P..NG.G. ...------- ---------- -......... ..........
CSP70_Brazil-BA     .......... ...D...... .......... .D........ D......... ..P..NG.G. ...------- ---------- -......... ..........
CSP71_Brazil-BA     .......... ...D...... .......... .D........ D......... ..P..NG.G. ...------- ---------- -......... ..........
CSP72_Brazil-BA     .......... ...D...... .......... .D........ .......... ..P..NG.G. ...------- ---------- -......... ..........
CSP74_Brazil-BA     .......... ...D...... .......... .D........ .......... ..P..NG.G. ...------- ---------- R......... ..........
CSP75_Brazil-BA     .......... .......... .......... .......... .......... ..P....... ...------- ---------- R......... ..........
CSP76_Brazil-BA     .......... ...D...... .......... .......... .........D ..P..NG.G. ...------- ---------- -......... ..........
CSP80_Brazil-BA     .......... ...D...... .......... .D........ D........D ..P..NG.G. ...------- ---------- -......... ..........
CSP82_Brazil-BA     .......... ...D...... .......... .......... D......... ..P..NG.G. ...------- ---------- -......... ..........
CSP84_Brazil-BA     ....A..... ...D...... ..A....... .......... .......... ..P..NG.G. ...------- ---------- -......... ..........
CSP87_Brazil-BA     ....A..... ...D...... ..A....... .......... .......... .......... ...------- ---------- -......... ..........
CSP88_Brazil-BA     .......... ...D...... .......... .D........ D........D ..P....... ...------- ---------- -......... ..........
CSP89_Brazil-BA     ....A..... ...D...... ..A....... .......... D......... ..P..NG.G. ...------- ---------- -......... ..........
CSP90_Brazil-BA     ....A..... ...D...... .......... .......... .......... ..P..NG.G. ...------- ---------- -......... ..........
CSP91_Brazil-BA     .......... ...D...... .......... .......... .......... ..P..NG.G. ...------- ---------- -......... ..........
CSP92_Brazil-BA     ....A..... .......... ..A....... .......... .......... ..P....... ...------- ---------- -......... ..........
CSP93_Brazil-BA     .......... ...D...... .......... .D........ D....----- ---------- ---------- ---------- -......... ..........
CSP95_Brazil-BA     .......... .......... .......... .......... .......... ..P....... .P.------- ---------- -......... ..........
CSP97_Brazil-BA     ....A..... .......... .......... .......... .......S.. ..P..NG.G. ...------- ---------- -......... ..........
CSP98_Brazil-BA     ....A..... ...D...... ..A....... .......... .......... ..P..NG.G. ...------- ---------- -......... ..........
CSP101_Brazil-BA    ....A..... ...D...... ..A....... .D........ .......... ..P..NG.G. ...------- ---------- -......... ..........
CSP105_Brazil-BA    .......... .......... .......... .......... .......... ..P....... ...------- ---------- -......... ..........
CSP106_Brazil-BA    .......... ...D...... .......... .......... .....----- ---------- ---------- ---------- -......... ..........
CSP107_Brazil-BA    .......... .......... ..A....... .......... .......S.. ..P....... .P.------- ---------- -......... ..........
CSP108_Brazil-BA    .......... ...D...... .......... .D........ D......... ..P....... ...------- ---------- -......... ..........
CSP109_Brazil-BA    ....A..... .......... ..A....... .......... .......... ..P....... .P.GDRAAGQ PAGNGAGGQA A......... ..........
CSP110_Brazil-BA    .......... ...D...... .......... .D........ .......... ..P....... .P.------- ---------- -......... ..........
CSP115_Brazil-BA    .......... ...D...... ..A....... .......... .......... ..P....... ...------- ---------- -......... ..........
CSP116_Brazil-BA    .......... .......... .......... .......... .......... .......... ...------- ---------- -......... ..........
```

```
CSP118_Brazil-BA         .......... ...D...... .......... .D........ D........D ..P..NG.G. ...------- ---------- -......... ..........
CSP120_Brazil-BA         ....A..... ...D...... ..A....... .......... .......... ..P..NG.G. ...------- ---------- -......... ..........
CSP121_Brazil-BA         ....A..... ...D...... ..A....... .......... .......... ..P..NG.G. ...------- ---------- -......... ..........
                                210        220        230        240        250        260        270        280        290        300
                         ....|....| ....|....| ....|....| ....|....| ....|....| ....|....| ....|....| ....|....| ....|....| ....|....|
PVX_119355_|_Salvador 1  GDRADGQPAG DRAAGQPAGD RADGQPAGDR AAGQPAGDRA AGQPAGDRAA GQAAGDRAAG QAA------- ---------- -GGNAGGQGQ NNEGANAPNE
CSP122_Brazil-BA         ....A..... ...D...... ..A....... .......... .......... ..P..NG.G. ...------- ---------- -......... ..........
CSP123_Brazil-BA         .......... ...D...... .......... .......... .......... ..P....... .P.------- ---------- -......... ..........
CSP124_Brazil-BA         ....A..... .......... .......... .......... .......S.V ..P..NG.G. ...------- ---------- -......... ..........
CSP125_Brazil-BA         .......... ...D...... .......... .......... .......S.. ..P.------ ---------- ---------- -......... ..........
CSP126_Brazil-BA         .......... ...D...... ..A....... .......... .......... ..P...S.G. ...------- ---------- -......... ..........
CSP127_Brazil-BA         .......... .......... .......... .......... .......... ..P..N.... .P.GNGAGGQ AA-------- -......... ..........
CSP128_Brazil-BA         .......... .......... .......... .......... .......... .......... ...GDRAAGQ AA-------- -......... ..........
CSP133_Brazil-BA         .......... .......... .......... .......... .......... .......... ...GDRAAGQ AA-------- -......... ..........
CSP135_Brazil-BA         .......... ...DV..... .......... .D........ D........V ..P..NG.G. ...------- ---------- -R........ ..........
CSP142_Brazil-BA         .......... .......... .......... .......... .........V ..P..NG.G. ...------- ---------- -......... ..........
CSP163_Brazil-BA         .......... ...D...... .......... .D........ D...------ ---------- ---------- ---------- -......... ..........
CSP167_Brazil-BA         .......... .......... .......... .......... .......... ..P....... .P.GDRAAGQ AA-------- -......... ..........
CSP170_Brazil-BA         .......... .......... .......... .......... .......... ..P....... ...------- ---------- -......... ..........
CSP174_Brazil-BA         .......... .......... .......... .......... .......... ..P....... .P.GDRAAGQ AA-------- -......... ..........
CSP179_Brazil-BA         ....A..... ...D...... ..A....... .......... .......... ..P..NG.G. ...------- ---------- -......... ..........
CSP181_Brazil-BA         ....A..... ...D...... ..A....... .......... .......... ..P..NG.G. ...------- ---------- -......... ..........
CSP182_Brazil-BA         ....A..... ...D...... ..A....... .......... .......... ..P..NG.G. ...------- ---------- -......... ..........
CSP186_Brazil-BA         .......... .......... .......... .......... .......... ..P....... .P.GNGAGGQ AA-------- -......... ..........
CSP190_Brazil-BA         .......... .......... .......... .......... .......... ..P....... .P.GDRAAGQ PAGNGAGGQA A......... ..........
CSP192_Brazil-BA         ....A..... .......... ..A....... .......... .......... ..P....... .P.GDRAAGQ PAGNGAGGQA A......... ..........
CSP193_Brazil-BA         .......... ...D...... .......... .......... D........D ..P..NG.G. ...------- ---------- -......... ..........
CSP194_Brazil-BA         ....A..... ...D...... ..A....... .......... .......... ..P..NG.G. ...------- ---------- -......... ..........
CSP195_Brazil-BA         .......... ....V..... .......... .......... .......... ..P..N.... .P.------- ---------- -......... ..........
CSP196_Brazil-BA         .......... .......... .......... .......... .......... ..P....... ...------- ---------- -......... ..........
CSP197_Brazil-BA         .......... ...D...... .......... .D........ D........D ..P..NG.G. ...------- ---------- -......... ..........
CSP198_Brazil-BA         .......... .......... .......... .......... .......... ..P....... .P.------- ---------- -......... ..........
CSP204_Brazil-BA         .......... .......... .......... .......... .......... ..P....... .P.------- ---------- -......... ..........
CSP210_Brazil-BA         ....A..... ...D...... ..A....... .......... .......... ..P..NG.G. ...------- ---------- -......... ..........
CSP213_Brazil-BA         .......... ...D...... .......... .D........ D........D ..P....... ...------- ---------- -......... ..........
CSP214_Brazil-BA         .......... ...D...... .......... .D........ D........D ..P..NG.G. ...------- ---------- -......... ..........
CSP222_Brazil-BA         .......... .......... .......... .......... .......... ..P....... .P.GNGAGGQ AA-------- -......... ..........
CSP224_Brazil-BA         ....A..... ...D...... ..A....... .......... .......... ..P..NG.G. ...------- ---------- -......... ..........
CSP226_Brazil-BA         ....A..... ...D...... ..A....... .......... .......... ..P...S.G. ...------- ---------- -......... ..........
CSP227_Brazil-BA         .......... ...D...... .......... .D........ .........V ..P..NG.G. ...------- ---------- -......... ..........
CSP231_Brazil-BA         ....A..... ...D...... ..A....... .......... .......... ..P..NG.G. ...------- ---------- -......... ..........
CSP232_Brazil-BA         ....A..... ...D...... ..A....... .......... .......... ..P..NG.G. ...------- ---------- -......... ..........
CSP235_Brazil-BA         .......... ...D...... ..A....... .......... .......... ..P..NG.G. ...------- ---------- -......... ..........
CSP237_Brazil-BA         .......... ...D...... .......... .......... .......... ..P....... ...------- ---------- -......... ..........
CSP238_Brazil-BA         ....A..... ...D...... ..A....... .......... .......... ..P..NG.G. ...------- ---------- -......... ..........
CSP239_Brazil-BA         .......... ...D...... .......... .......... .......... ..P..NG.G. ...------- ---------- -......... ..........
CSP240_Brazil-BA         .......... .......... .......... .D........ D........D ..P..NG.G. ...------- ---------- -......... ..........
CSP241_Brazil-BA         .......... .......... .......... .......... .........D ..P..NG.G. ...------- ---------- -......... ..........
CSP245_Brazil-BA         ....A..... .......... ..A....... .......... .......... ..P..NG.G. ...------- ---------- -......... ..........
CSP249_Brazil-BA         .......... ...D...... .......... .......... .......... ..P....... .P.------- ---------- -......... ..........
CSP57_Brazil-AF          .......... .......... .......... .......... .........V ..P..NG.G. ...------- ---------- -......... ..........
CSP64_Brazil-AF          .......... .......... .......... .......... .......... ..P....... .P.------- ---------- -......... ..........
CSP67_Brazil-AF          .......... ...D...... .......... .D........ D........D ..P..NG.G. ...------- ---------- -......... ..........
CSP81_Brazil-AF          ....A..... .......... ..A....... .......... .......... ..P..NG.G. ...------- ---------- -......... ..........
CSP86_Brazil-AF          ....A..... ....V..... .......... .......... D......S.V ..P..NG.G. ...------- ---------- -R........ ..........
CSP96_Brazil-AF          .......... .......... ..A....... .......... .......... ..P....... .P.------- ---------- -......... ..........
CSP100_Brazil-AF         ....A..... .......... ..A....... .......... .......... ..P....... .P.GNGAGGQ AA-------- -......... ..........
```

```
CSP102_Brazil-AF           ....A..... .......... ..A....... .D........ .......... ..P....... .P.------- ---------- -......... ..........
CSP103_Brazil-AF           ....A..... .......... ..A....... .......... .......... ..P....... .P.------- ---------- -......... ..........
CSP104_Brazil-AF           ....A..... .......... .......... .......... .......... ..P....... ...------- ---------- -......... ..........
                                    210        220        230        240        250        260        270        280        290        300
                           ....|....| ....|....| ....|....| ....|....| ....|....| ....|....| ....|....| ....|....| ....|....| ....|....|
PVX_119355_|_Salvador 1    GDRADGQPAG DRAAGQPAGD RADGQPAGDR AAGQPAGDRA AGQPAGDRAA GQAAGDRAAG QAA------- ---------- -GGNAGGQGQ NNEGANAPNE
CSP130_Brazil-AF           ....A..... .......... ..A....... .......... .......... ..P..NG.G. ...------- ---------- -......... ..........
CSP132_Brazil-AF           ....A..... .......... ..A....... .......... .......... ..P....... .P.GDRAAGQ PAGNGAGGQA A......... ..........
CSP134_Brazil-AF           .......... ...D...... .......... .......... .......S.V ..P....... ...------- ---------- -......... ..........
CSP136_Brazil-AF           ....A..... .......... ..A....... .......... .......... ..P....... .P.GDRAAGQ PAGNGAGGQA A......... ..........
CSP137_Brazil-AF           .......... ...D...... .......... .......... .......S.. ..P..NG.G. ...------- ---------- -......... ..........
CSP138_Brazil-AF           ....A..... .......... ..A....... .......... .......... ..P....... .P.GDRAAGQ PAGNGAGGQA A......... ..........
CSP139_Brazil-AF           ....A..... .......... ..A....... .......... .......S.. ..P..NG.G. ...------- ---------- -R........ ..........
CSP140_Brazil-AF           ....A..... .......... ..A....... .......... .......... ..P....... .P.GDRAAGQ PAGNGAGGQA A......... ..........
CSP143_Brazil-AF           ....A..... .......... ..A....... .......... .......... ..P....... .P.GDRAAGQ PAGNGAGGQA A......... ..........
CSP144_Brazil-AF           ....A..... .......... ..A....... .......... .......... ..P....... ...GDRAAGQ PAGNGAGGQA A......... ..........
CSP145_Brazil-AF           ....A..... .......... ..A....... .......... .......... ..P....... .P.GDRAAGQ PAGNGAGGQA A......... ..........
CSP146_Brazil-AF           ....A..... ....V..... ..A....... .......... .......... ..P...S.G. ...GDRAAGQ PAGNGAGGQA A......... ..........
CSP147_Brazil-AF           ....A..... ....V..... ..A....... .......... .......... ..P....... .P.GDRAAGQ PAGNGAGGQA A......... ..........
CSP148_Brazil-AF           ....A..... ....V..... ..A....... .......... .......... ..P....... .P.GDRAAGQ PAGNGAGGQA A......... ..........
CSP149_Brazil-AF           ....A..... .......... ..A....... .......... .......... .......... ...GDRAAGQ PAGNGAGGQA A......... ..........
CSP150_Brazil-AF           ....A..... .......... ..A....... .......... .......... .....NG.G. ...------- ---------- -......... ..........
CSP151_Brazil-AF           ....A..... .......... ..A....... .......... .......... ..P....... ...GDRAAGQ PAGNGAGGQA A......... ..........
CSP152_Brazil-AF           ....A..... .......... ..A....... .......... .......... ..P....... ...------- ---------- -......... ..........
CSP153_Brazil-AF           ....A..... .......... ..A....... .......... .......... ..P....... ...------- ---------- -......... ..........
CSP155_Brazil-AF           .......... .......... .......... .......... .........V ..P..NG.G. ...------- ---------- -......... ..........
CSP156_Brazil-AF           ....A..... .......... ..A....... .......... .......... ..P..NG.G. ...------- ---------- -......... ..........
CSP159_Brazil-AF           ....A..... .......... ..A....... .......... .......... .......... ...GDRAAGQ PAGNGAGGQA A......... ..........
CSP160_Brazil-AF           ....A..... .......... ..A....... .......... .......... ..P...S.G. ...GDRAAGQ PAGNGAGGQA A......... ..........
CSP161_Brazil-AF           ....A..... .......... ..A....... .......... .......... ..P....... ...GDRAAGQ PAGNGAGGQA A......... ..........
CSP162_Brazil-AF           ....A..... .......... .......... .......... .......... ..P....... .P.------- ---------- -R........ ..........
CSP171_Brazil-AF           ....A..... ...D...... ..A....... .......... .......... ..P....... .P.GDRAAGQ PAGNGAGGQA A......... ..........
CSP172_Brazil-AF           .......... .......... .......... .......... .......... ..P....... ...------- ---------- -......... ..........
CSP173_Brazil-AF           ....A..... ...D...... ..A....... .......... .......... ..P..NG.G. ...------- ---------- -......... ..........
CSP175_Brazil-AF           ....A..... .......... ..A....... .......... .......... ..P....... .P.GDRAAGQ PAGNGAGGQA A......... ..........
CSP176_Brazil-AF           ....A..... .......... ..A....... .......... .......... .......... ...GDRAAGQ PAGNGAGGQA A......... ..........
CSP177_Brazil-AF           ....A..... .......... ..A....... .......... .......... ..P....... .P.GDRAAGQ PAGNGAGGQA A......... ..........
CSP180_Brazil-AF           ....A..... .......... ..A....... .......... .......... ..P....... .P.GDRAAGQ PAGNGAGGQA A......... ..........
CSP183_Brazil-AF           ....A..... .......... ..A....... .......... .......... ..P....... .P.GDRAAGQ PAGNGAGGQA A......... ..........
CSP184_Brazil-AF           ....A..... ...D...... ..A....... .......... .......... ..P..NG.G. ...------- ---------- -......... ..........
CSP185_Brazil-AF           ....A..... .......... ..A....... .......... .......... ..P....... .P.GDRAAGQ PAGNGAGGQA A......... ..........
CSP187_Brazil-AF           ....A..... .......... ..A....... .......... .......... ..P..NG.G. ...------- ---------- -......... ..........
CSP188_Brazil-AF           ....A..... .......... ..A....... .......... .......... ..P...S.G. ...------- ---------- -......... ..........
CSP189_Brazil-AF           .......... ...DV..... .......... .......... .......... ..P..N.... .P.------- ---------- -......... ..........
CSP191_Brazil-AF           ....A..... ...D...... ..A....... .......... .......... ..P..NG.G. ...------- ---------- -......... ..........
CSP200_Brazil-AF           ....A..... .......... ..A....... .......... .......... ..P....... .P.------- ---------- -......... ..........
CSP201_Brazil-AF           .......... .......... ..A....... .......... .......... ..P..NG.G. ...------- ---------- -......... ..........
CSP202_Brazil-AF           ....A..... .......... ..A....... .......... .......... ..P....... ...------- ---------- -......... ..........
CSP203_Brazil-AF           ....A..... .......... ..A....... .......... .......... ..P....... ...------- ---------- -......... ..........
CSP205_Brazil-AF           .......... ...D...... .......... .D........ .......... ..P....... .P.------- ---------- -......... ..........
CSP206_Brazil-AF           .......... .......... ..A....... .......... .......... ..P....... .P.GDRAAGQ PAGNGAGGQA A......... ..........
CSP207_Brazil-AF           ....A..... .......... ..A....... .......... .......... ..P....... ...GDRAAGQ PAGNGAGGQA A......... ..........
CSP209_Brazil-AF           ....A..... ...DV..... .......... .D........ .......... ..P..NG.G. ...GDRAAGQ PAGNGAGGQA A......... ..........
CSP211_Brazil-AF           ....A..... .......... ..A....... .......... .........V ..P..NG.G. ...------- ---------- -......... ..........
CSP212_Brazil-AF           ....A..... .......... ..A....... .......... .........V ..P..NG.G. ...------- ---------- -......... ..........
CSP215_Brazil-AF           ....A..... .......... ..A....... .......... .......... ..P....... ...GDRAAGQ PAGNGAGGQA A......... ..........
CSP217_Brazil-AF           ....A..... .......... ..A....... .......... .......... ..P..NG.G. ...------- ---------- -......... ..........
```

```
CSP218_Brazil-AF            ....A..... ...D...... ..A....... .......... .......... ..P....... .P.------- ---------- -......... ..........
CSP219_Brazil-AF            ....A..... .......... ..A....... .......... .......... ..P..NG.G. ...------- ---------- -......... ..........
CSP220_Brazil-AF            .......... ...D...... .......... .......... .........V ..P..NG.G. ...------- ---------- -......... ..........
                                   210        220        230        240        250        260        270        280        290        300
                            ....|....| ....|....| ....|....| ....|....| ....|....| ....|....| ....|....| ....|....| ....|....| ....|....|
PVX_119355_|_Salvador 1     GDRADGQPAG DRAAGQPAGD RADGQPAGDR AAGQPAGDRA AGQPAGDRAA GQAAGDRAAG QAA------- ---------- -GGNAGGQGQ NNEGANAPNE
CSP221_Brazil-AF            .......... .......... .......... .......... .........V ..P..NG.G. ...------- ---------- -......... ..........
CSP223_Brazil-AF            .......... ...D...... .......... .......... .......... ..P..NG.G. ...------- ---------- -......... ..........
CSP233_Brazil-AF            .......... ...D...... .......... .D........ D........D ..P..NG.G. ...------- ---------- -......... ..........
CSP234_Brazil-AF            ....A..... .......... .......... .......... D........D ..P..NG.G. ...------- ---------- -......... ..........
CSP242_Brazil-AF            .......... .......... .......... .D........ .......... ..P..NG.G. ...------- ---------- -......... ..........
CSP247_Brazil-AF            ....A..... .......... .......... .......... .......... ..P..NG.G. ...------- ---------- -......... ..........
CSP250_Brazil-AF            ....A..... .......... ..A....... .......... .......... ..P....... .P.------- ---------- -......... ..........
CSP77_Angola                ....A..... .......... .......... .......... .......... ..P....... .P.------- ---------- -......... ..........
CSP94_Angola                .......... ...D...... .......... .D........ D........D ..P..NG.G. ...------- ---------- -......... ..........
CSP119_Angola               ....A..... ....V..... .......... .......... .......S.D ..P....... ...------- ---------- -......... ..........
CSP228_Burkina_Faso         .......... ...D...... ..A....... .D........ D......S.V ..P..NG.G. ...------- ---------- -......... ..........
CSP229_Ivory_Coast          ....A..... .......... .......... .......... .........V ..P...S.G. ...------- ---------- -......... ..........
CSP113_Suriname             .......... ...D...... .......... .D........ D...------ ---------- ---------- ---------- -......... ..........
CSP117_French_Guiana        ....A..... ...D...... ..A....... .D........ D........D ..P..NS.G. .P.------- ---------- -......... ..........
CSP129_Haiti                ....A..... .......... .......... .......... .......... ..P....... ...------- ---------- -R........ ..........
CSP165_Colombia             .......... .......... .......... .......... .......... ..P....... .P.------- ---------- -......... ..........
CSP168_Peru                 .......... ...D...... .......... .......... .......... ..P....... ...------- ---------- -......... ..........
CSP169_Venezuela            .......... .......... .......... .D........ ....------ ---------- ---------- ---------- -......... ..........
CSP216_Venezuela            .......... ...D...... ..A....... .......... .......... ..P..NG.G. ...------- ---------- -......... ..........

                                   310        320        330        340        350        360
                            ....|....| ....|....| ....|....| ....|....| ....|....| ....|....| ..
PVX_119355_|_Salvador 1     KSVKEYLDKV RATVGTEWTP CSVTCGVGVR VRRRVNAANK KPEDLTLNDL ETDVCTMDKC AG
CSP1_Brazil-BA              .......... .......... .......... .......... .......... .......... ..
CSP2_Brazil-BA              .......N.. .......... .......... .......... .......... .......... ..
CSP3_Brazil-BA              .......N.. .......... .......... .......... .......... .......... ..
CSP4_Brazil-BA              .......N.. .......... .......... .......... .......... .......... ..
CSP5_Brazil-BA              .......... .......... .......... .......... .......... .......... ..
CSP6_Brazil-BA              .......... .......... .......... .......... .......... .......... ..
CSP7_Brazil-BA              .......... .......... .......... .......... .......... .......... ..
CSP8_Brazil-BA              .......... .......... .......... .......... .......... .......... ..
CSP9_Brazil-BA              .......... .......... .......... .......... .......... .......... ..
CSP10_Brazil-BA             .......... .......... .......... .......... .......... .......... ..
CSP11_Brazil-BA             .......... .......... .......... .......... .......... .......... ..
CSP12_Brazil-BA             .......... .......... .......... .......... .......... .......... ..
CSP13_Brazil-BA             .......... .......... .......... .......... .......... .......... ..
CSP14_Brazil-BA             .......... .......... .......... .......... .......... .......... ..
CSP15_Brazil-BA             .......... .......... .......... .......... .......... .......... ..
CSP16_Brazil-BA             .......N.. .......... .......... .......... .......... .......... ..
CSP18_Brazil-BA             .......N.. .......... .......... .......... .......... .......... ..
CSP19_Brazil-BA             .......... .......... .......... .......... .......... .......... ..
CSP20_Brazil-BA             .......... .......... .......... .......... .......... .......... ..
CSP21_Brazil-BA             .......... .......... .......... .......... .......... .......... ..
CSP22_Brazil-BA             .......... .......... .......... .......... .......... .......... ..
CSP23_Brazil-BA             .......... .......... .......... .......... .......... .......... ..
CSP24_Brazil-BA             .......... .......... .......... .......... .......... .......... ..
CSP25_Brazil-BA             .......... .......... .......... .......... .......... .......... ..
CSP26_Brazil-BA             .......... .......... .......... .......... .......... .......... ..
CSP27_Brazil-BA             .......... .......... .......... .......... .......... .......... ..
CSP28_Brazil-BA             .......... .......... .......... .......... .......... .......... ..
CSP29_Brazil-BA             .......... .......... .......... .......... .......... .......... ..
```

```
CSP30_Brazil-BA            .......... .......... .......... .......... .......... .......... ..
CSP31_Brazil-BA            .......... .......... .......... .......... .......... .......... ..
CSP32_Brazil-BA            .......... .......... .......... .......... .......... .......... ..
                                  310        320        330        340        350        360
                           ....|....| ....|....| ....|....| ....|....| ....|....| ....|....| ..
PVX_119355_|_Salvador 1    KSVKEYLDKV RATVGTEWTP CSVTCGVGVR VRRRVNAANK KPEDLTLNDL ETDVCTMDKC AG
CSP33_Brazil-BA            .......... .......... .......... .......... .......... .......... ..
CSP34_Brazil-BA            .......... .......... .......... .......... .......... .......... ..
CSP35_Brazil-BA            .......... .......... .......... .......... .......... .......... ..
CSP36_Brazil-BA            .......... .......... .......... .......... .......... .......... ..
CSP37_Brazil-BA            .......... .......... .......... .......... .......... .......... ..
CSP38_Brazil-BA            .......... .......... .......... .......... .......... .......... ..
CSP40_Brazil-BA            .......... .......... .......... .......... .......... .......... ..
CSP41_Brazil-BA            .......... .......... .......... .......... .......... .......... ..
CSP42_Brazil-BA            .......N.. .......... .......... .......... .......... .......... ..
CSP43_Brazil-BA            .......... .......... .......... .......... .......... .......... ..
CSP44_Brazil-BA            .......... .......... .......... .......... .......... .......... ..
CSP45_Brazil-BA            .......... .......... .......... .......... .......... .......... ..
CSP47_Brazil-BA            .......N.. .......... .......... .......... .......... .......... ..
CSP48_Brazil-BA            .......... .......... .......... .......... .......... .......... ..
CSP49_Brazil-BA            .......... .......... .......... .......... .......... .......... ..
CSP50_Brazil-BA            .......... .......... .......... .......... .......... .......... ..
CSP52_Brazil-BA            .......... .......... .......... .......... .......... .......... ..
CSP53_Brazil-BA            .......... .......... .......... .......... .......... .......... ..
CSP54_Brazil-BA            .......... .......... .......... .......... .......... .......... ..
CSP55_Brazil-BA            .......... .......... .......... .......... .......... .......... ..
CSP56_Brazil-BA            .......... .......... .......... .......... .......... .......... ..
CSP58_Brazil-BA            .......... .......... .......... .......... .......... .......... ..
CSP59_Brazil-BA            .......... .......... .......... .......... .......... .......... ..
CSP60_Brazil-BA            .......... .......... .......... .......... .......... .......... ..
CSP61_Brazil-BA            .......... .......... .......... .......... .......... .......... ..
CSP62_Brazil-BA            .......... .......... .......... .......... .......... .......... ..
CSP63_Brazil-BA            .......... .......... .......... .......... .......... .......... ..
CSP65_Brazil-BA            .......... .......... .......... .......... .......... .......... ..
CSP66_Brazil-BA            .......... .......... .......... .......... .......... .......... ..
CSP69_Brazil-BA            .......... .......... .......... .......... .......... .......... ..
CSP70_Brazil-BA            .......... .......... .......... .......... .......... .......... ..
CSP71_Brazil-BA            .......... .......... .......... .......... .......... .......... ..
CSP72_Brazil-BA            .......... .......... .......... .......... .......... .......... ..
CSP74_Brazil-BA            .......N.. .......... .......... .......... .......... .......... ..
CSP75_Brazil-BA            .......... .......... .......... .......... .......... .......... ..
CSP76_Brazil-BA            .......... .......... .......... .......... .......... .......... ..
CSP80_Brazil-BA            .......... .......... .......... .......... .......... .......... ..
CSP82_Brazil-BA            .......... .......... .......... .......... .......... .......... ..
CSP84_Brazil-BA            .......... .......... .......... .......... .......... .......... ..
CSP87_Brazil-BA            .......... .......... .......... .......... .......... .......... ..
CSP88_Brazil-BA            .......... .......... .......... .......... .......... .......... ..
CSP89_Brazil-BA            .......... .......... .......... .......... .......... .......... ..
CSP90_Brazil-BA            .......... .......... .......... .......... .......... .......... ..
CSP91_Brazil-BA            .......... .......... .......... .......... .......... .......... ..
CSP92_Brazil-BA            .......... .......... .......... .......... .......... .......... ..
CSP93_Brazil-BA            .......... .......... .......... .......... .......... .......... ..
CSP95_Brazil-BA            .......... .......... .......... .......... .......... .......... ..
CSP97_Brazil-BA            .......... .......... .......... .......... .......... .......... ..
CSP98_Brazil-BA            .......... .......... .......... .......... .......... .......... ..
CSP101_Brazil-BA           .......... .......... .......... .......... .......... .......... ..
CSP105_Brazil-BA           .......... .......... .......... .......... .......... .......... ..
```

```
CSP106_Brazil-BA            .......... .......... .......... .......... .......... .......... ..
CSP107_Brazil-BA            .......... .......... .......... .......... .......... .......... ..
CSP108_Brazil-BA            .......... .......... .......... .......... .......... .......... ..
                                   310        320        330        340        350        360
                            ....|....| ....|....| ....|....| ....|....| ....|....| ....|....| ..
PVX_119355_|_Salvador 1     KSVKEYLDKV RATVGTEWTP CSVTCGVGVR VRRRVNAANK KPEDLTLNDL ETDVCTMDKC AG
CSP109_Brazil-BA            .......... .......... .......... .......... .......... .......... ..
CSP110_Brazil-BA            .......... .......... .......... .......... .......... .......... ..
CSP115_Brazil-BA            .......... .......... .......... .......... .......... .......... ..
CSP116_Brazil-BA            .......... .......... .......... .......... .......... .......... ..
CSP118_Brazil-BA            .......... .......... .......... .......... .......... .......... ..
CSP120_Brazil-BA            .......... .......... .......... .......... .......... .......... ..
CSP121_Brazil-BA            .......... .......... .......... .......... .......... .......... ..
CSP122_Brazil-BA            .......... .......... .......... .......... .......... .......... ..
CSP123_Brazil-BA            .......... .......... .......... .......... .......... .......... ..
CSP124_Brazil-BA            .......... .......... .......... .......... .......... .......... ..
CSP125_Brazil-BA            .......... .......... .......... .......... .......... .......... ..
CSP126_Brazil-BA            .......... .......... .......... .......... .......... .......... ..
CSP127_Brazil-BA            .......... .......... .......... .......... .......... .......... ..
CSP128_Brazil-BA            .......... .......... .......... .......... .......... .......... ..
CSP133_Brazil-BA            .......... .......... .......... .......... .......... .......... ..
CSP135_Brazil-BA            .......... .......... .......... .......... .......... .......... ..
CSP142_Brazil-BA            .......... .......... .......... .......... .......... .......... ..
CSP163_Brazil-BA            .......... .......... .......... .......... .......... .......... ..
CSP167_Brazil-BA            .......... .......... .......... .......... .......... .......... ..
CSP170_Brazil-BA            .......... .......... .......... .......... .......... .......... ..
CSP174_Brazil-BA            .......... .......... .......... .......... .......... .......... ..
CSP179_Brazil-BA            .......... .......... .......... .......... .......... .......... ..
CSP181_Brazil-BA            .......... .......... .......... .......... .......... .......... ..
CSP182_Brazil-BA            .......... .......... .......... .......... .......... .......... ..
CSP186_Brazil-BA            .......... .......... .......... .......... .......... .......... ..
CSP190_Brazil-BA            .......... .......... .......... .......... .......... .......... ..
CSP192_Brazil-BA            .......... .......... .......... .......... .......... .......... ..
CSP193_Brazil-BA            .......... .......... .......... .......... .......... .......... ..
CSP194_Brazil-BA            .......... .......... .......... .......... .......... .......... ..
CSP195_Brazil-BA            .......N.. .......... .......... .......... .......... .......... ..
CSP196_Brazil-BA            .......... .......... .......... .......... .......... .......... ..
CSP197_Brazil-BA            .......... .......... .......... .......... .......... .......... ..
CSP198_Brazil-BA            .......... .......... .......... .......... .......... .......... ..
CSP204_Brazil-BA            .......... .......... .......... .......... .......... .......... ..
CSP210_Brazil-BA            .......... .......... .......... .......... .......... .......... ..
CSP213_Brazil-BA            .......... .......... .......... .......... .......... .......... ..
CSP214_Brazil-BA            .......... .......... .......... .......... .......... .......... ..
CSP222_Brazil-BA            .......... .......... .......... .......... .......... .......... ..
CSP224_Brazil-BA            .......... .......... .......... .......... .......... .......... ..
CSP226_Brazil-BA            .......... .......... .......... .......... .......... .......... ..
CSP227_Brazil-BA            .......... .......... .......... .......... .......... .......... ..
CSP231_Brazil-BA            .......... .......... .......... .......... .......... .......... ..
CSP232_Brazil-BA            .......... .......... .......... .......... .......... .......... ..
CSP235_Brazil-BA            .......... .......... .......... .......... .......... .......... ..
CSP237_Brazil-BA            .......... .......... .......... .......... .......... .......... ..
CSP238_Brazil-BA            .......... .......... .......... .......... .......... .......... ..
CSP239_Brazil-BA            .......... .......... .......... .......... .......... .......... ..
CSP240_Brazil-BA            .......... .......... .......... .......... .......... .......... ..
CSP241_Brazil-BA            .......... .......... .......... .......... .......... .......... ..
CSP245_Brazil-BA            .......... .......... .......... .......... .......... .......... ..
CSP249_Brazil-BA            .......... .......... .......... .......... .......... .......... ..
```

```
CSP57_Brazil-AF            .......... .......... .......... .......... .......... .......... ..
CSP64_Brazil-AF            .......... .......... .......... .......... .......... .......... ..
CSP67_Brazil-AF            .......... .......... .......... .......... .......... .......... ..
                                   310        320        330        340        350        360
                           ....|....| ....|....| ....|....| ....|....| ....|....| ....|....| ..
PVX_119355_|_Salvador 1    KSVKEYLDKV RATVGTEWTP CSVTCGVGVR VRRRVNAANK KPEDLTLNDL ETDVCTMDKC AG
CSP81_Brazil-AF            .......... .......... .......... .......... .......... .......... ..
CSP86_Brazil-AF            .......... .......... .......... .......... .......... .......... ..
CSP96_Brazil-AF            .......... .......... .......... .......... .......... .......... ..
CSP100_Brazil-AF           .......... .......... .......... .......... .......... .......... ..
CSP102_Brazil-AF           .......... .......... .......... .......... .......... .......... ..
CSP103_Brazil-AF           .......... .......... .......... .......... .......... .......... ..
CSP104_Brazil-AF           .......... .......... .......... .......... .......... .......... ..
CSP130_Brazil-AF           .......... .......... .......... .......... .......... .......... ..
CSP132_Brazil-AF           .......... .......... .......... .......... .......... .......... ..
CSP134_Brazil-AF           .......... .......... .......... .......... .......... .......... ..
CSP136_Brazil-AF           .......... .......... .......... .......... .......... .......... ..
CSP137_Brazil-AF           .......... .......... .......... .......... .......... .......... ..
CSP138_Brazil-AF           .......... .......... .......... .......... .......... .......... ..
CSP139_Brazil-AF           .......... .......... .......... .......... .......... .......... ..
CSP140_Brazil-AF           .......... .......... .......... .......... .......... .......... ..
CSP143_Brazil-AF           .......... .......... .......... .......... .......... .......... ..
CSP144_Brazil-AF           .......... .......... .......... .......... .......... .......... ..
CSP145_Brazil-AF           .......... .......... .......... .......... .......... .......... ..
CSP146_Brazil-AF           .......... .......... .......... .......... .......... .......... ..
CSP147_Brazil-AF           .......... .......... .......... .......... .......... .......... ..
CSP148_Brazil-AF           .......... .......... .......... .......... .......... .......... ..
CSP149_Brazil-AF           .......... .......... .......... .......... .......... .......... ..
CSP150_Brazil-AF           .......... .......... .......... .......... .......... .......... ..
CSP151_Brazil-AF           .......... .......... .......... .......... .......... .......... ..
CSP152_Brazil-AF           .......... .......... .......... .......... .......... .......... ..
CSP153_Brazil-AF           .......... .......... .......... .......... .......... .......... ..
CSP155_Brazil-AF           .......... .......... .......... .......... .......... .......... ..
CSP156_Brazil-AF           .......... .......... .......... .......... .......... .......... ..
CSP159_Brazil-AF           .......... .......... .......... .......... .......... .......... ..
CSP160_Brazil-AF           .......... .......... .......... .......... .......... .......... ..
CSP161_Brazil-AF           .......... .......... .......... .......... .......... .......... ..
CSP162_Brazil-AF           .......... .......... .......... .......... .......... .......... ..
CSP171_Brazil-AF           .......... .......... .......... .......... .......... .......... ..
CSP172_Brazil-AF           .......... .......... .......... .......... .......... .......... ..
CSP173_Brazil-AF           .......... .......... .......... .......... .......... .......... ..
CSP175_Brazil-AF           .......... .......... .......... .......... .......... .......... ..
CSP176_Brazil-AF           .......... .......... .......... .......... .......... .......... ..
CSP177_Brazil-AF           .......... .......... .......... .......... .......... .......... ..
CSP180_Brazil-AF           .......... .......... .......... .......... .......... .......... ..
CSP183_Brazil-AF           .......... .......... .......... .......... .......... .......... ..
CSP184_Brazil-AF           .......... .......... .......... .......... .......... .......... ..
CSP185_Brazil-AF           .......N.. .......... .......... .......... .......... .......... ..
CSP187_Brazil-AF           .......... .......... .......... .......... .......... .......... ..
CSP188_Brazil-AF           .......... .......... .......... .......... .......... .......... ..
CSP189_Brazil-AF           .......N.. .......... .......... .......... .......... .......... ..
CSP191_Brazil-AF           .......... .......... .......... .......... .......... .......... ..
CSP200_Brazil-AF           .......... .......... .......... .......... .......... .......... ..
CSP201_Brazil-AF           .......... .......... .......... .......... .......... .......... ..
CSP202_Brazil-AF           .......... .......... .......... .......... .......... .......... ..
CSP203_Brazil-AF           .......... .......... .......... .......... .......... .......... ..
CSP205_Brazil-AF           .......... .......... .......... .......... .......... .......... ..
```

```
CSP206_Brazil-AF           .......... .......... .......... .......... .......... .......... ..
CSP207_Brazil-AF           .......... .......... .......... .......... .......... .......... ..
CSP209_Brazil-AF           .......... .......... .......... .......... .......... .......... ..
                                  310        320        330        340        350        360
                           ....|....| ....|....| ....|....| ....|....| ....|....| ....|....| ..
PVX_119355_|_Salvador 1    KSVKEYLDKV RATVGTEWTP CSVTCGVGVR VRRRVNAANK KPEDLTLNDL ETDVCTMDKC AG
CSP211_Brazil-AF           .......... .......... .......... .......... .......... .......... ..
CSP212_Brazil-AF           .......... .......... .......... .......... .......... .......... ..
CSP215_Brazil-AF           .......... .......... .......... .......... .......... .......... ..
CSP217_Brazil-AF           .......... .......... .......... .......... .......... .......... ..
CSP218_Brazil-AF           .......N.. .......... .......... .......... .......... .......... ..
CSP219_Brazil-AF           .......... .......... .......... .......... .......... .......... ..
CSP220_Brazil-AF           .......... .......... .......... .......... .......... .......... ..
CSP221_Brazil-AF           .......... .......... .......... .......... .......... .......... ..
CSP223_Brazil-AF           .......... .......... .......... .......... .......... .......... ..
CSP233_Brazil-AF           .......... .......... .......... .......... .......... .......... ..
CSP234_Brazil-AF           .......... .......... .......... .......... .......... .......... ..
CSP242_Brazil-AF           .......... .......... .......... .......... .......... .......... ..
CSP247_Brazil-AF           .......N.. .......... .......... .......... .......... .......... ..
CSP250_Brazil-AF           .......... .......... .......... .......... .......... .......... ..
CSP77_Angola               .......... .......... .......... .......... .......... .......... ..
CSP94_Angola               .......... .......... .......... .......... .......... .......... ..
CSP119_Angola              .......... .......... .......... .......... .......... .......... ..
CSP228_Burkina_Faso        .......... .......... .......... .......... .......... .......... ..
CSP229_Ivory_Coast         .......... .......... .......... .......... .......... .......... ..
CSP113_Suriname            .......... .......... .......... .......... .......... .......... ..
CSP117_French_Guiana       .......... .......... .......... .......... .......... .......... ..
CSP129_Haiti               .......... .......... .......... .......... .......... .......... ..
CSP165_Colombia            .......... .......... .......... .......... .......... .......... ..
CSP168_Peru                .......... .......... .......... .......... .......... .......... ..
CSP169_Venezuela           .......... .......... .......... .......... .......... .......... ..
CSP216_Venezuela           .......... .......... .......... .......... .......... .......... ..
```

The amino acid sequence of the *pvcsp* amplified region. The highlight in grey is the central region motif. PVX_119355: ID of the reference gene in Plasmodb.org; BA: Brazilian Amazon region; AF: Atlantic Forest region.
